# Hierarchical Interfacial Engineering of Boehmite‐Coated Separators via Silane Functionalization and Electron‐Beam Irradiation

**DOI:** 10.1002/smll.75250

**Published:** 2026-08-16

**Authors:** Md Amir Sohel, Sungwoo Kim, Seunguk Cheon, Sung Oh Cho

**Affiliations:** ^1^ Department of Nuclear and Quantum Engineering Korea Advanced Institute of Science and Technology (KAIST) Daejeon Republic of Korea

**Keywords:** boehmite, ceramic‐coated separator, electron‐beam irradiation, interfacial engineering, lithium‐ion battery

## Abstract

Interfacial instability at the ceramic–polymer interface in ceramic‐coated separators remains a critical challenge, as weak adhesion can cause coating delamination and compromise lithium‐ion battery safety and electrochemical performance. Here, we report a dual strategy combining (3‐aminopropyl)triethoxysilane (APTES)‐functionalized boehmite (AlOOH) with electron‐beam (EB) irradiation to reinforce ceramic‐coated high‐density polyethylene (HDPE) separators. APTES promotes coupling between AlOOH and the poly(acrylic acid) binder, while EB irradiation strengthens the interfacial network. APTES functionalization increases tensile strength from 136.13 to 167.78 MPa and elongation at break from 37.52% to 72.84%. Irradiation at 50 kGy further raises tensile strength to 171.72 MPa while retaining 66.28% elongation. The combined treatments increase peel strength from 118.27 to 257.68 N m^−^^1^. At 150°C and 160°C, shrinkage decreases from 83.23% and 95.21% for HDPE to 2.46% and 9.52% at 50 kGy, and 0.70% and 7.74% at 75 kGy, respectively. Ionic conductivity increases from 0.166 ± 0.005 mS cm^−1^ for HDPE to 0.635 ± 0.030 and 0.823 ± 0.028 mS cm^−1^ at 50 and 75 kGy, respectively. The 75 kGy separator achieves a Li^+^ transference number of 0.68% and 98.97% capacity retention after 200 cycles. This approach enables thermally stable, mechanically robust separators through coupled chemical and radiation‐driven interface engineering.

## Graphical abstract

A hierarchically reinforced ceramic–coated separator is developed by integrating APTES‐functionalized boehmite nanoparticles with EB‐induced radical formation and subsequent coupling. This synergistic interface engineering enables significantly improved mechanical integrity and interfacial stability. The separator delivers exceptional mechanical strength and adhesion, suppressed thermal shrinkage, and fast Li^+^ transport, enabling outstanding cycling stability and advancing the development of thermally stable, high‐performance lithium‐ion battery separators.

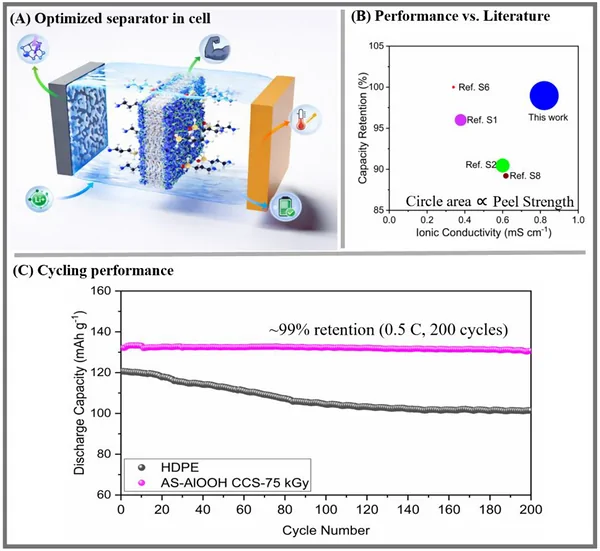

## 1 Introduction

Lithium‐ion batteries (LIBs) underpin modern energy storage technologies, powering applications ranging from portable electronics to electric vehicles and grid‐scale systems, owing to their high energy density and long cycle life [1, 2, 3, 4]. However, increasing energy density has intensified safety challenges, particularly those associated with thermal runaway, internal short circuits, and structural instability under elevated temperatures or abusive conditions [3, 4, 5, 6, 7, 8, 9]. In this context, the separator plays a pivotal role in maintaining both electrochemical performance and intrinsic safety by preventing electronic contact between electrodes while enabling efficient ionic transport [9, 10, 11]. Commercial polyolefin separators, such as polyethylene (PE) and polypropylene (PP), dominate the current battery market due to their chemical stability, mechanical robustness, and low cost [2, 4, 8, 9]. Nevertheless, their intrinsic hydrophobicity, low surface polarity, and severe thermal shrinkage at elevated temperatures—typically above 120°C for PE [12] and 150°C for PP [13]—significantly limit their safety margins in high‐energy systems [2, 11, 14]. To address these limitations, ceramic‐coated separators (CCSs) have been widely explored as an effective strategy to integrate thermally stable inorganic frameworks with polymer substrates, thereby improving thermal resistance, mechanical integrity, and electrolyte wettability [12, 15, 16, 17, 18, 19, 20, 21, 22, 23, 24, 25, 26, 27, 28, 29, 30, 31, 32, 33, 34]. Among ceramic materials, Al_2_O_3_ [15, 16, 17, 18, 19, 20, 23, 34] has been extensively explored for its excellent thermal stability and mechanical strength, although its relatively low hydroxyl density often necessitates additional surface modification to achieve sufficient adhesion and electrolyte wettability. In contrast, SiO_2_ [21, 22] offers abundant hydroxyl groups and improved electrolyte affinity but suffers from inferior mechanical robustness and particle aggregation. Other ceramics, including TiO_2_ [23, 24] and ZrO_2_ [25, 26], exhibit high thermal stability but are often limited by challenges related to cost, density, or processing scalability. Aluminum oxyhydroxide (AlOOH, boehmite) represents a compelling alternative, combining high thermal stability (>450°C) with a hydroxyl‐rich surface that promotes strong interfacial interactions with polymer binders [27, 28, 29, 30]. Moreover, its compatibility with aqueous processing routes offers practical and environmentally benign fabrication advantages [35]. Despite these advantages, the performance of CCSs remains fundamentally governed by the interfacial chemistry between ceramic nanoparticles and polymer binders. Poly(acrylic acid) (PAA) [28, 31, 32, 36, 37], with its abundant carboxyl functionalities, is widely employed as an aqueous binder; however, insufficient particle–binder interfacial bonding often leads to coating delamination and mechanical degradation under thermal and electrochemical stress, ultimately compromising separator reliability. Silane coupling agents provide a molecular‐level strategy to bridge inorganic and organic phases [21, 28, 38, 39]. In particular, (3‐aminopropyl)triethoxysilane (APTES) is well suited for AlOOH–PAA systems [21, 28, 39]. APTES can form stable Si─O─Al bonds with boehmite surfaces while the terminal amine (─NH_2_) groups interact with the PAA binder, thereby enabling the formation of chemically integrated interfacial networks. However, silane‐mediated coupling alone is insufficient to fully stabilize the ceramic–polymer interface under severe mechanical and thermal conditions. Electron‐beam (EB) irradiation offers an additional route to modify polymeric separator and binder components through radiation‐induced radical formation and subsequent recoupling or crosslinking [39, 40, 41, 42, 43, 44]. EB processing can promote radical‐mediated crosslinking within polyolefin substrates and polymer binders without chemical initiators, thereby enhancing dimensional stability and mechanical integrity while preserving porosity. Nevertheless, EB irradiation alone does not address weak ceramic–polymer interfacial bonding. Therefore, a key challenge remains: how to simultaneously engineer strong chemical coupling and robust interfacial reinforcement within ceramic‐coated separators to achieve durable interfacial integration. To date, the synergistic coupling of silane‐functionalized boehmite with EB‐induced crosslinking for hierarchical interface engineering has not been systematically explored.

Here, we report a hierarchical interfacial engineering strategy that integrates APTES functionalized boehmite nanoparticles with controlled EB irradiation to construct mechanically robust and thermally stable AlOOH‐coated HDPE separators. The combination of APTES–mediated chemical coupling and EB irradiation–induced radical reactions reinforces the ceramic–polymer framework, improving interfacial adhesion, thermal dimensional stability, and ion transport. By systematically correlating interfacial chemistry with mechanical, thermal, and electrochemical properties, this work elucidates the design principles of molecular‐level interface engineering and provides a scalable pathway towards safer and high‐performance lithium‐ion battery separators.

## 2 Results and Discussion

### 2.1 APTES Functionalization of AlOOH NPs

The hydroxyl (–OH)‐rich surface of pristine AlOOH nanoparticles promotes strong interparticle hydrogen bonding, leading to aggregation [45], which destabilizes the coating slurry and results in non‐uniform deposition on the HDPE substrate. To overcome these limitations, a controlled hydrolysis–condensation strategy [46] was employed to graft APTES onto the AlOOH surface. Figure 1a schematically illustrates the evolution of the surface chemistry of AlOOH NPs before and after APTES functionalization. Pristine boehmite possesses a high density of –OH groups from its layered oxyhydroxide lattice, which serve as anchoring sites for silanol species generated during APTES hydrolysis. Subsequent condensation reactions form covalent Al–O–Si linkages, yielding a conformal organic layer terminated with outward‐oriented amine (─NH_2_) groups on the nanoparticle surface. This surface chemistry modification suppresses particle–particle interactions, improves colloidal stability, and enhances compatibility with polymer matrices via chemically active –NH_2_ functionalities [47]. The FESEM image (Figure 1b) reveals plate‐like and blocky particles with sharp facets, characteristic of crystalline boehmite. TEM analysis (Figure 1c) confirms anisotropic platelet morphologies with lateral dimensions of 400–500 nm. High‐magnification TEM (Figure 1d) reveals a uniform APTES‐derived surface layer with a thickness of ∼21.3 nm. This relatively thick layer is attributed to an APTES‐derived multilayer containing an oligomeric polysiloxane network, formed through Al–O–Si anchoring and simultaneous Si–O–Si self‐condensation under the HCl assisted functionalization conditions. High‐resolution TEM (Figure 1e) shows well‐defined lattice fringes, indicating that the crystalline framework is preserved after functionalization, while the corresponding SAED pattern confirms the polycrystalline yet highly ordered nature of the AlOOH core. HAADF–STEM combined with EDS mapping (Figure 1f) provides compositional evidence of surface‐selective grafting. Aluminum and oxygen are uniformly distributed throughout the particle interior. In contrast, silicon and nitrogen, originating from the silane backbone and terminal –NH_2_ groups, are preferentially enriched at the particle periphery. This confirms that APTES is localized on the surface without bulk incorporation. FTIR spectroscopy (Figure 1g) further verifies the surface chemistry of AlOOH after APTES functionalization (AS‐AlOOH). Pristine AlOOH exhibits the characteristic vibrations of its hydroxyl‐rich oxyhydroxide lattice. Two prominent bands at 3268.80 and 3082.82 cm^−1^ correspond to –OH stretching modes of surface and structural –OH groups. In the fingerprint region, absorptions at 1147.93, 1072.24 and 730.90 cm^−1^ are assigned to Al─O / Al─O─H lattice vibrations, including Al─O stretching and Al─O─H bending modes, confirming the crystalline oxyhydroxide structure of AlOOH. AS‐AlOOH exhibits these lattice features together with silane‐related bands, indicating the formation of an organic–inorganic hybrid interphase. New absorption bands at 1608.85 and 1505.19 cm^−1^ are attributed to ─NH_2_ bending vibrations, while a band at 1222.66 cm^−1^ corresponds to Si─C/C─N stretching. Additionally, the bands at 1067.20 and 943.99 cm^−1^ are assigned to Si─O─Al and Si─O─Si stretching modes, respectively. The Si─O─Al band supports covalent anchoring of APTES to the AlOOH surface, whereas the Si─O─Si band indicates APTES self‐condensation and formation of an oligomeric polysiloxane network. To quantify the extent of APTES functionalization, thermogravimetric analysis (TGA) was performed (Figure S1). Compared with pristine AlOOH, AS‐AlOOH exhibits an additional mass loss in the intermediate temperature region attributable to thermal decomposition of the grafted organic silane layer. Based on the differential weight loss, the grafting amount is estimated to be approximately 7.62 wt.%, confirming the formation of a substantial organic layer. Comparable TGA behavior and grafting levels have been reported for APTES‐functionalized AlOOH prepared under acidic conditions [46], supporting the reliability of the present functionalization strategy. X‐ray diffraction (XRD) analysis (Figure 1h) further verifies that the silane treatment under acidic condition does not perturb the bulk crystal structure. Both pristine AlOOH and AS‐AlOOH exhibit diffraction patterns indexed to orthorhombic boehmite (AlOOH; JCPDS 00‐021‐1307), with identical peak positions and no detectable secondary phases, confirming the preservation of phase purity and long‐range crystallographic order. The absence of additional reflections attributable to APTES supports its amorphous and surface‐confined nature. Collectively, these microscopic and spectroscopic results demonstrate that APTES functionalization forms a conformal –NH_2_‐rich interphase while maintaining the structural integrity of the crystalline AlOOH core, providing surface characteristics conducive to reduced particle aggregation in the slurry and uniform coating formation on the HDPE separator.

**FIGURE 1:**
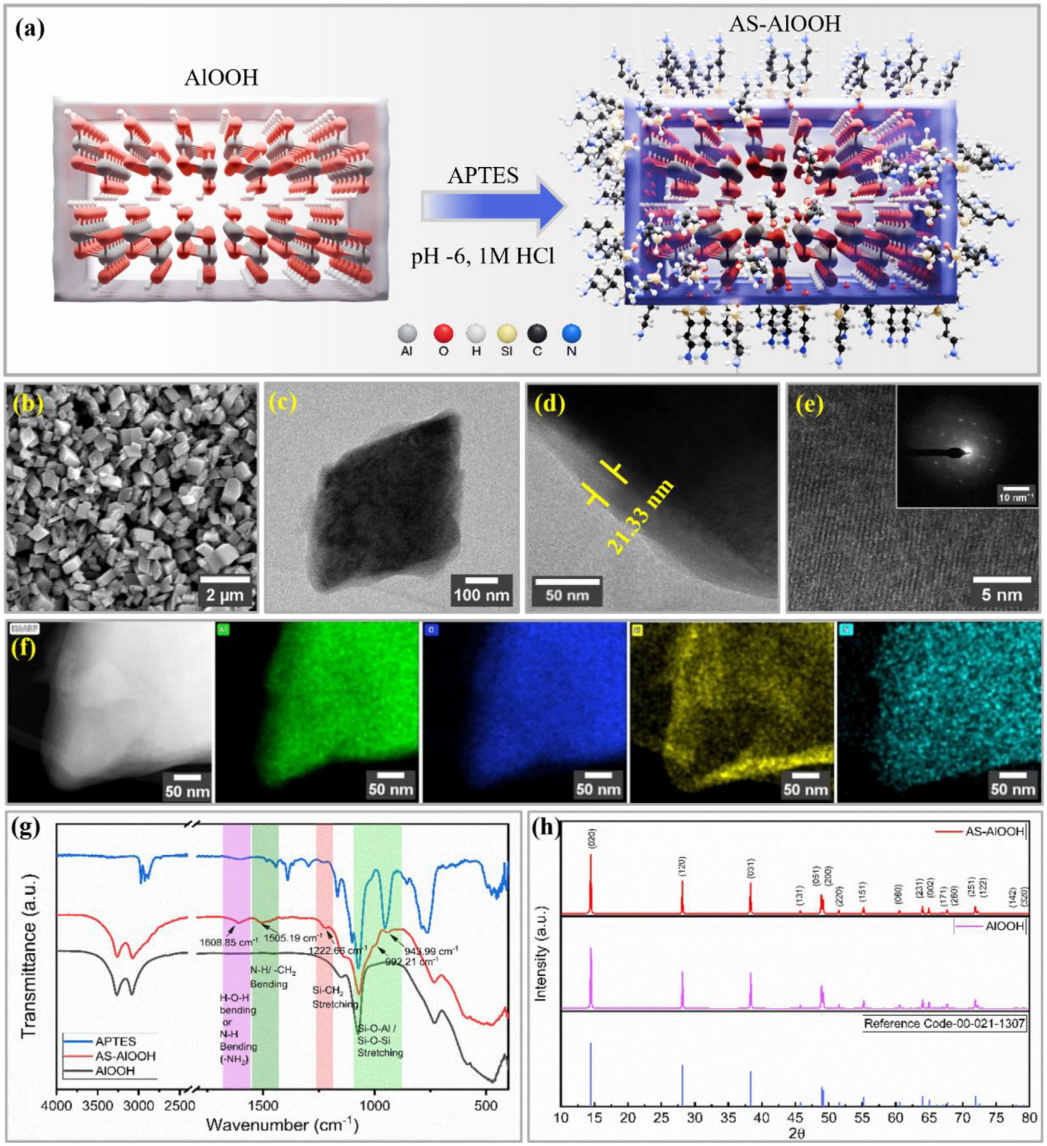
(a) Schematic illustration of the surface functionalization of AlOOH with APTES. (b) FESEM image showing the morphology of AS‐AlOOH nanoparticles. (c) TEM image of AS‐AlOOH. (d,e) HRTEM images revealing the APTES layer and lattice fringes with the corresponding SAED pattern (inset). (f) STEM‐EDS elemental mapping confirms the uniform distribution of Al, O, Si, and N in AS‐AlOOH. (g) FTIR spectra of APTES, pristine AlOOH, and AS‐AlOOH. (h) XRD patterns of AlOOH and AS‐AlOOH with reference to JCPDS card No. 00‐021‐1307.

### 2.2 Separator Properties

Figure 2a schematically illustrates the structural configurations of the HDPE separator and the ceramic‐coated separator (CCS), while Figure 2b depicts the proposed interfacial interactions within the ceramic‐coated and EB‐irradiated separators. In the AlOOH CCS, the AlOOH nanoparticle surface is enriched with terminal hydroxyl (–OH) groups, whereas the poly(acrylic acid) (PAA) binder contains abundant carboxyl (–COOH) functionalities. Consequently, interfacial adhesion is expected to be governed by hydrogen bonding between Al─OH and ─COOH groups, together with weak van der Waals interactions with the chemically inert HDPE substrate, resulting in limited interfacial compatibility and relatively weak adhesion. Following surface functionalization with (3‐aminopropyl)triethoxysilane (APTES) to prepare the AS‐AlOOH CCS, Al–O–Si linkages are formed through condensation between the hydrolyzed silanol groups and the hydroxylated AlOOH surface, while silane self‐condensation may also produce Si–O–Si structures. Simultaneously, the outward‐oriented amine (–NH_2_) groups can interact strongly with the PAA binder through acid–base ionic interactions involving –NH_3_^+^ ···COO^–^ ion pairs, in addition to the hydrogen bonding interactions between the residual surface –OH groups of AS‐AlOOH and the –COOH groups of PAA. These combined chemical interactions are expected to enhance the adhesion between the AS‐AlOOH nanoparticles and the PAA binder and may improve the coating integrity. Upon subsequent EB irradiation, high‐energy electrons may induce bond cleavage and hydrogen abstraction within HDPE, PAA, and the organosilane layer anchored to the AlOOH surface. These processes can generate carbon‐ and oxygen‐centered radicals within the interfacial organic components. The generated radicals may undergo recombination, which may lead to crosslinking within HDPE and PAA, and interfacial coupling across the HDPE–PAA, AS‐AlOOH–PAA, and HDPE–AS‐AlOOH interfaces. Collectively, these bonding pathways may produce a more interconnected interfacial structure that anchors the AS‐AlOOH‐containing coating to the HDPE substrate, which may in turn improve coating adhesion and interfacial integrity. In addition, similar interfacial functionalization strategies are widely recognized to influence ion‐transport kinetics in advanced separators [33].

**FIGURE 2:**
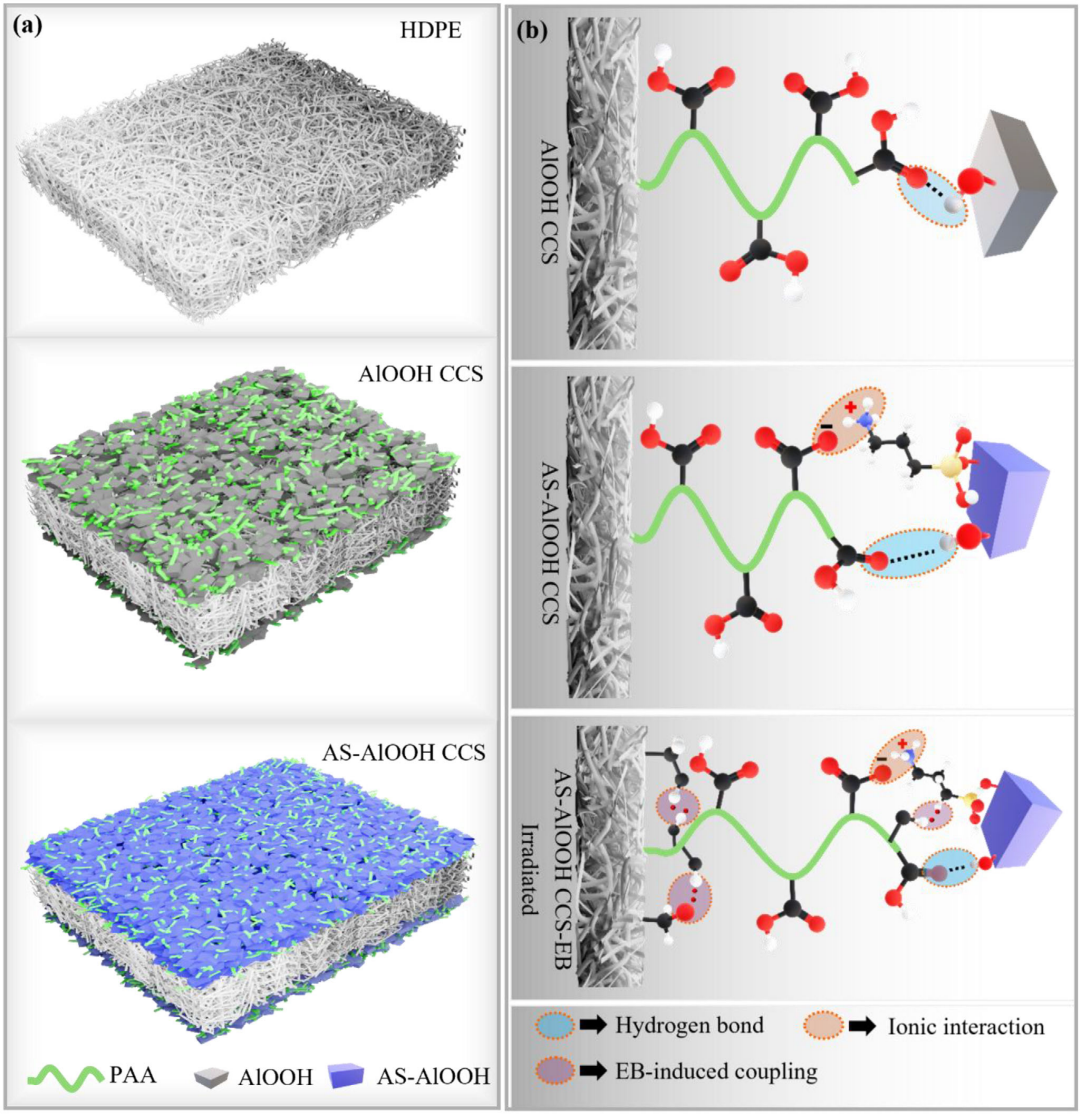
Schematic illustration of (a) the structural evolution from HDPE to AS‐AlOOH CCS separators and (b) the proposed interfacial interactions, highlighting hydrogen bonding, ionic interactions and potential EB‐induced radical‐mediated coupling.

Figure 3a–h presents the surface and cross‐sectional FESEM images of pristine HDPE and ceramic‐coated separators with corresponding elemental analysis. The pristine HDPE separator (Figure 3a) exhibits the characteristic fibrillar and interconnected porous morphology typical of stretched polyolefin membranes. After coating with pristine AlOOH nanoparticles (AlOOH CCS, Figure 3b), the AlOOH nanoparticles are deposited on the HDPE surface; however, pronounced nanoparticle agglomeration results in incomplete surface coverage, leaving localized regions of the underlying HDPE substrate exposed. This heterogeneous morphology arises from the high surface polarity and strong self‐association tendency of hydroxyl (–OH)‐rich AlOOH nanoparticles, which limit dispersion stability and weaken interfacial contact with the HDPE substrate. In contrast, the AS‐AlOOH CCS (Figure 3c) displays a compact, highly uniform ceramic layer with minimal aggregation, indicating substantially improved dispersion and interfacial compatibility imparted by APTES functionalization. Elemental mapping of Si (Figure 3d) in the AS‐AlOOH CCS reveals a homogeneous surface distribution, and together with the complementary C, O, Al, and Si mappings (Figure S2), confirms the uniform distribution of AS‐AlOOH nanoparticles throughout the coating layer. Cross‐sectional FESEM images of the AlOOH CCS separator (Figure 3e) reveal ceramic coatings on both sides of the HDPE substrate; however, the coating layer appears rough with localized thickness variations, attributable to AlOOH nanoparticle agglomeration and limited interfacial compatibility between the AlOOH nanoparticles and the nonpolar HDPE surface. In contrast, the cross‐section image of the AS‐AlOOH CCS separator (Figure 3f) shows a uniform and conformal ceramic coating with a tightly integrated interface on both sides of the HDPE substrate. The separator thickness increases from ∼10 µm for pristine HDPE to ∼13 µm after coating, corresponding to ∼1.5 µm ceramic layers on each side while preserving the porous framework. The cross‐sectional EDS mappings of C and Si for the AS‐AlOOH CCS separator are presented in Figure 3g,h, respectively. The carbon mapping (Figure 3g) shows a continuous signal concentrated in the central region corresponding to the HDPE substrate, confirming the preservation of the polymer framework after coating. In contrast, the silicon mapping (Figure 3h) shows two symmetric bands along the outer regions of the separator, corresponding to the ceramic coating layers. The absence of Si in the core region confirms that the AS‐AlOOH nanoparticles remain predominantly localized within the coating layers rather than penetrating the HDPE substrate. The continuous and uniform Si distribution further indicates homogeneous nanoparticle dispersion and consistent coating thickness, confirming the formation of a well‐defined multilayer separator architecture.

**FIGURE 3:**
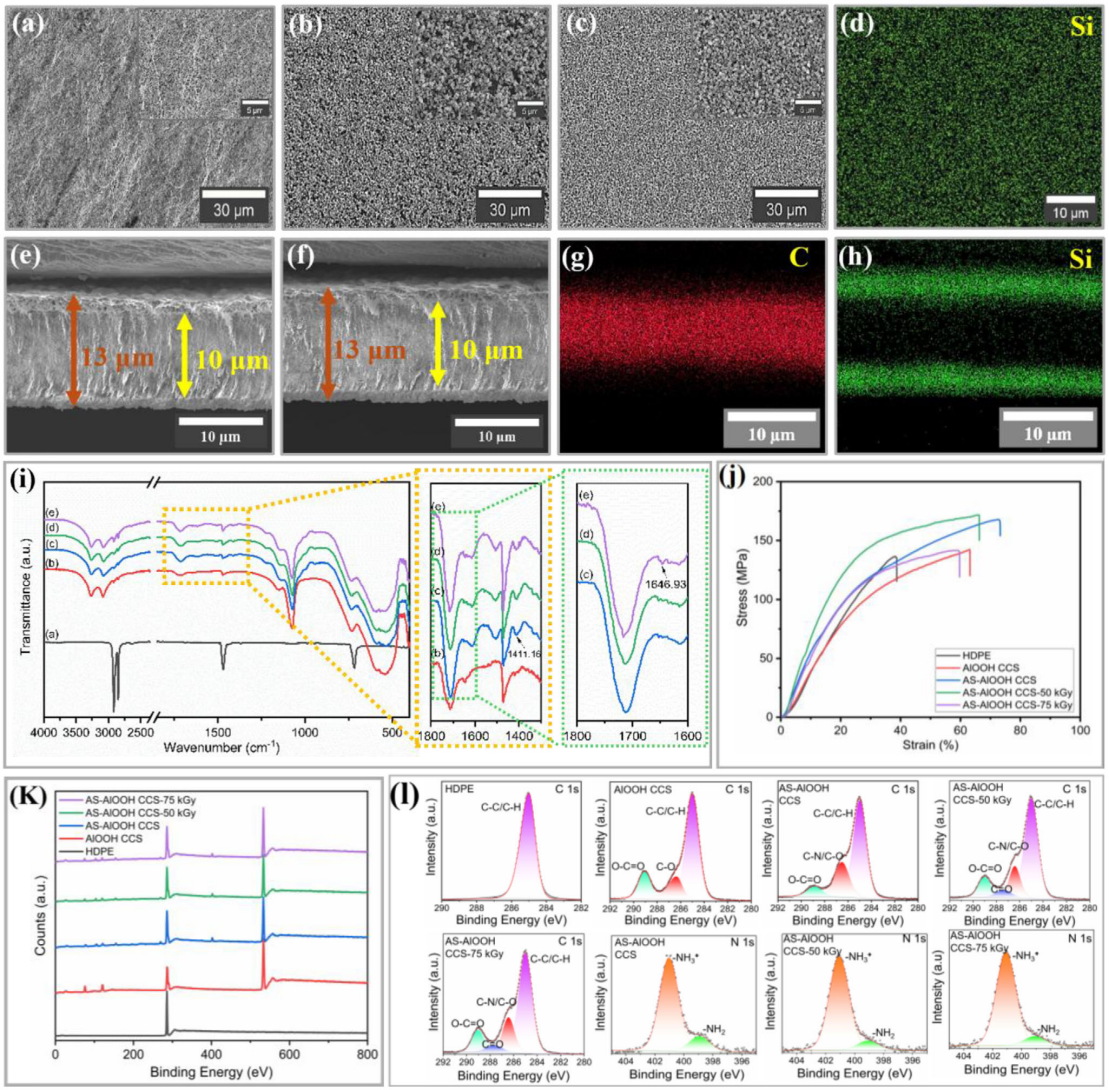
Surface morphology, elemental distribution, chemical structure, and mechanical properties of separators. (a–c) FESEM surface images of HDPE, AlOOH CCS, and AS‐AlOOH CCS (insets: higher magnification). (d) EDS Si mapping of AS‐AlOOH CCS. (e,f) Cross‐sectional FESEM images of AlOOH CCS and AS‐AlOOH CCS. (g,h) Cross‐sectional EDS mappings of C and Si for AS‐AlOOH CCS. (i) FTIR spectra of HDPE, AlOOH CCS, AS‐AlOOH CCS, AS‐AlOOH CCS‐50 kGy, and AS‐AlOOH CCS‐75 kGy. (j) Stress–strain curves of HDPE, AlOOH CCS, AS‐AlOOH CCS, AS‐AlOOH CCS‐50 kGy, and AS‐AlOOH CCS‐75 kGy. (k) XPS survey spectra of the separators. (l) High resolution C 1s and N 1s spectra showing different chemical environments in the separators.

To verify the proposed interfacial bonding mechanisms, FTIR spectroscopy was used to probe chemical interactions in the AlOOH CCS, AS‐AlOOH CCS, AS‐AlOOH CCS‐50 kGy, and AS‐AlOOH CCS‐75 kGy separators. The spectra of pristine HDPE and modified separators are shown in Figure 3i. The HDPE spectrum (curve a) exhibits characteristic vibrations, including asymmetric and symmetric ─CH_2_─ stretching at 2915.40 and 2848.39 cm^−1^, –CH_2_ scissoring at 1468.55 cm^−1^, and crystalline rocking at 716.91 cm^−1^, reflecting its semi‐crystalline hydrocarbon backbone and nonpolar surface chemistry. The AlOOH CCS spectrum (curve b) displays additional features associated with hydroxylated AlOOH and the PAA binder, particularly a carbonyl stretching band at 1713.95 cm^−1^, shifted from 1694 cm^−1^ in pristine PAA (Figure S3a). This shift indicates hydrogen bonding between the –COOH groups of PAA and the –OH groups of AlOOH, supporting interfacial interactions within the ceramic‐binder network [48, 49]. For the AS‐AlOOH CCS separator, the spectrum (curve c) additionally displays Si─O─Si/Si─O─Al vibrations together with amine (–NH_2_) related bands at 1614.63 and 1505.67 cm^−1^, and a carboxylate feature near 1411.17 cm^−1^. These signatures indicate acid–base complexation (–NH_3_^+^–COO^−^) between the –NH_2_ groups on the AS‐AlOOH surface and the –COOH groups of PAA, consistent with previously reported spectroscopic signatures of acid‐base interactions in polyacid–amine complexes. Thus, in addition to the hydrogen bonding observed in AlOOH CCS, the AS‐AlOOH system introduces additional acid–base ionic coupling, yielding a more strongly integrated ceramic–polymer interface. Following electron‐beam irradiation, the overall spectral features remain largely unchanged, indicating structural stability of the ceramic–polymer framework. However, a weak band at 1646.93 cm^−1^ appears for AS‐AlOOH CCS‐75 kGy. Although this region may contain an amide I contribution, it can also include overlapping vibrations from adsorbed water, carboxylate species, and other carbonyl‐containing groups. Therefore, the appearance of this band is conservatively interpreted as indicating EB‐induced interfacial modifications, potentially involving the generation of radicals and their subsequent coupling at the ceramic‐polymer interfaces.

To obtain complementary surface‐sensitive information on the interfacial chemical environment, XPS survey spectra were analyzed, as shown in Figure 3k, while the corresponding high‐resolution C 1s and N 1s spectra are presented in Figure 3l. The peak‐fitting parameters for C 1s and N 1s spectra are summarized in Tables S1 and S2, respectively. The HDPE separator exhibits only a dominant C–C/C–H component at 285.00 eV, reflecting its hydrocarbon structure and nonpolar surface chemistry. The AlOOH CCS separator exhibits additional C–O and O–C═O components at 286.35 and 289.06 eV, with relative contributions of 15.95% and 16.68%, respectively, confirming the presence of the PAA‐containing coating [48]. The AS‐AlOOH CCS separator exhibited a component approximately at 286 eV. The relative contribution of the component increases from 15.95% for C–O in the AlOOH CCS to 26.40% for the overlapping C–O/C–N contributions in the AS‐AlOOH CCS, accompanied by a slight shift in binding energy from 286.35 to 286.51 eV. This increase is mainly attributed to the additional C–N contribution from the APTES‐derived aminopropyl groups, supporting the incorporation of APTES and the resulting modification of the chemical environment at the AS‐AlOOH–PAA interface [46, 48]. In conjunction with the FTIR results, these observations support the successful incorporation of APTES and the establishment of an amine‐functionalized interfacial environment capable of interacting with the PAA binder. EB irradiation produces further changes in the C 1s spectra. The AS‐AlOOH CCS‐50 kGy and AS‐AlOOH CCS‐75 kGy separators exhibit additional C═O components at 287.39 and 287.66 eV, with relative contributions of 6.74% and 5.89%, respectively. Moreover, the O–C = O contribution increases from 8.27% in AS‐AlOOH CCS to 12.38% in AS‐AlOOH CCS‐50 kGy and 12.42% in AS‐AlOOH CCS‐75 kGy. Correspondingly, the C–C/C–H contribution decreases from 65.33% to 63.49% and 61.89%, respectively. These systematic changes indicate irradiation‐induced formation and redistribution of carbonyl‐ and carboxyl‐containing species within the interfacial organic components. These observed changes are consistent with radical‐mediated chemical reorganization expected during EB irradiation. In accordance with the proposed mechanism, subsequent radical recombination may contribute to polymer crosslinking and enhanced integration among the separator components [50, 51].

The N 1s spectrum of AS‐AlOOH CCS contains a dominant protonated –NH_3_^+^ component at 401.03 eV and a relatively smaller neutral –NH_2_ component at 398.93 eV, with relative contributions of 84.10% and 15.90%, respectively. The predominance of – NH_3_^+^ is primarily attributed to protonation of the APTES‐derived amine groups during the HCl assisted APTES functionalization of AlOOH [46, 49]. Following incorporation of the APTES‐functionalized AlOOH into the PAA‐containing coating, these protonated amines may participate in interfacial ionic interactions with carboxylate groups of PAA, forming –NH_3_^+^···^−^OOC– ion pairs [49]. After irradiation, the – NH_3_^+^ contribution increases to 89.86% in AS‐AlOOH CCS‐50 kGy and 93.61% in AS‐AlOOH CCS‐75 kGy, while the neutral –NH_2_ contribution decreases to 10.14% and 6.39%, respectively. These changes indicate that a progressively larger fraction of the amine groups participates in protonated or strongly interacting environments after irradiation. Together, the increased carbonyl and carboxyl contents and altered amine environment indicate EB‐induced oxidation, strengthened acid–base interactions, and interfacial chemical reorganization, with radical recombination potentially contributing to interfacial coupling [50, 51]. Collectively, the XPS results support key chemical features of the proposed mechanism illustrated in Figure 2b. The PAA‐derived oxygen‐containing components are consistent with the hydrogen‐bonded AlOOH–PAA environment, while APTES incorporation introduces aminopropyl functionalities and protonated amine environments capable of participating in acid–base ionic interactions with PAA. Following EB irradiation, the systematic redistribution of the carbon‐, oxygen‐, and nitrogen‐containing species provides evidence of interfacial chemical reorganization within the coating surface. Together with the FTIR results, these observations are consistent with irradiation‐enhanced interfacial integration and potential radical‐mediated coupling among the separator components.

Interfacial engineering combined with electron beam (EB) irradiation systematically modulates the mechanical integrity of the separators (Figure 3j). Pristine HDPE exhibits a tensile strength of 136.13 MPa and an elongation at break of 37.52%, reflecting ductile deformation primarily governed by lamellar slippage within the HDPE. After coating with AlOOH nanoparticles (AlOOH CCS), both tensile strength and elongation at break increase to 142.25 MPa and 62.94%, respectively. Although AlOOH is intrinsically brittle, the coating does not constitute a dense, monolithic ceramic film; rather, it comprises a porous particulate layer bound by PAA and supported by the ductile HDPE substrate. The limited coating thickness of approximately 1.5 µm on each side plays an important role in preserving separator deformability. This thin coating facilitates stress redistribution and reinforcement without imposing the severe deformation constraint typically associated with a thick, rigid ceramic layer. During tensile deformation, the PAA‐containing interparticle regions and porous coating structure can accommodate particle rearrangement, interfacial sliding, and progressive microcracking without immediately propagating failure into the HDPE substrate. Consequently, HDPE remains the primary load‐bearing and deformable component, allowing the multilayer separator to sustain greater deformation before complete fracture. Therefore, the increased elongation reflects the deformation behavior of the entire composite separator rather than the intrinsic ductility of the ceramic phase. Similar simultaneous increases in tensile strength and elongation at break have been reported for thin ceramic‐coated separators, where the ceramic–polymer interfacial layer enhances stress transfer and suppresses crack initiation and propagation [24, 52]. Further improvement is observed for the AS‐AlOOH CCS, which exhibits a tensile strength of 167.78 MPa and an elongation at break of 72.84%. This enhancement arises from APTES‐mediated interfacial coupling, which promotes uniform coverage of AS‐AlOOH nanoparticles and strengthens the interaction between the functionalized particle surface and the PAA binder. In particular, acid–base‐associated ionic interactions between protonated amine and carboxylate groups contribute to the formation of a more integrated ceramic–polymer interface, as supported by the combined FTIR and XPS results. The improved interfacial integrity facilitates effective stress transfer and delays interfacial debonding and crack propagation. EB irradiation further modifies the mechanical response of the AS‐AlOOH CCS separators. Irradiation at 50 kGy produces the highest tensile strength of 171.72 MPa while retaining a substantial elongation at break of 66.28%, indicating reinforcement of the polymer framework without severely restricting separator deformation. Increasing the dose to 75 kGy reduces the tensile strength and elongation at break of the AS‐AlOOH CCS‐75 kGy separator to 140.96 MPa and 59.56%, respectively, indicating irradiation‐induced embrittlement. The complete dose‐dependent tensile behavior of the AS‐AlOOH CCS separators over the range of 25–100 kGy is presented in Figure S4a,b. This trend is consistent with the dose‐dependent mechanical response of pristine HDPE shown in Figure S4c,d. At moderate irradiation doses, radical recombination and crosslinking can reinforce the polyolefin framework, resulting in a crosslinking‐dominated mechanical response. At higher doses, increasing network rigidity and chain‐scission reactions progressively reduce both strength and deformability. A similar EB dose‐dependent evolution of the mechanical properties of pristine HDPE separators has been reported previously [39].

The thermal shrinkage behavior of the separators is subsequently evaluated (Figure 4a,b). Pristine HDPE exhibits severe shrinkage, reaching 83.23% at 150°C and 95.21% at 160°C, due to thermal relaxation and collapse of the polyethylene lamellar structure. In contrast, the AlOOH CCS separator significantly suppresses shrinkage, resulting in 24.29% at 150°C and 40.14% at 160°C, indicating that the ceramic coating acts as a thermally stable framework that constrains deformation of the underlying HDPE substrate. Further reductions are observed for AS‐AlOOH CCS, which exhibits shrinkage values of 12.89% at 150°C and 19.32% at 160°C. This improvement reflects enhanced structural integrity arising from uniform nanoparticle distribution and APTES‐mediated interfacial coupling between the AS‐AlOOH nanoparticles and the PAA binder. EB irradiation markedly improves the dimensional stability. The AS‐AlOOH CCS‐50 kGy separator shows thermal shrinkage of 2.46% at 150°C and 9.52% at 160°C, while AS‐AlOOH CCS‐75 kGy exhibits the lowest shrinkage of 0.70% at 150°C and 7.74% at 160°C. This suppression is consistent with irradiation‐induced crosslinking within the HDPE/PAA framework, which can restrict polymer chain mobility at elevated temperatures. The progressive increase in gel fraction of the PAA film with EB dose (Figure S3b) supports crosslink network formation in PAA upon irradiation. EB dose‐dependent thermal shrinkage measurements (Figure S5a,b) further confirm that shrinkage decreases with irradiation dose, reaching a minimum at 75 kGy, but increases again at 100 kGy (7.39% at 150°C and 29.22% at 160°C) due to irradiation‐induced degradation. The 50 kGy condition provides an optimal balance between tensile strength and mechanical flexibility, whereas 75 kGy represents a higher crosslinking regime with minimal thermal shrinkage. In contrast, the 25 kGy dose induces only limited structural modification, whereas excessive irradiation at 100 kGy deteriorates both mechanical properties and high‐temperature dimensional stability. Accordingly, 50 and 75 kGy are selected as optimized EB irradiation doses for subsequent adhesion and electrochemical characterization. These results demonstrate that hierarchical interfacial engineering effectively stabilizes the separator architecture, thereby enhancing the thermal safety and dimensional reliability of ceramic‐coated separators under elevated temperatures.

**FIGURE 4:**
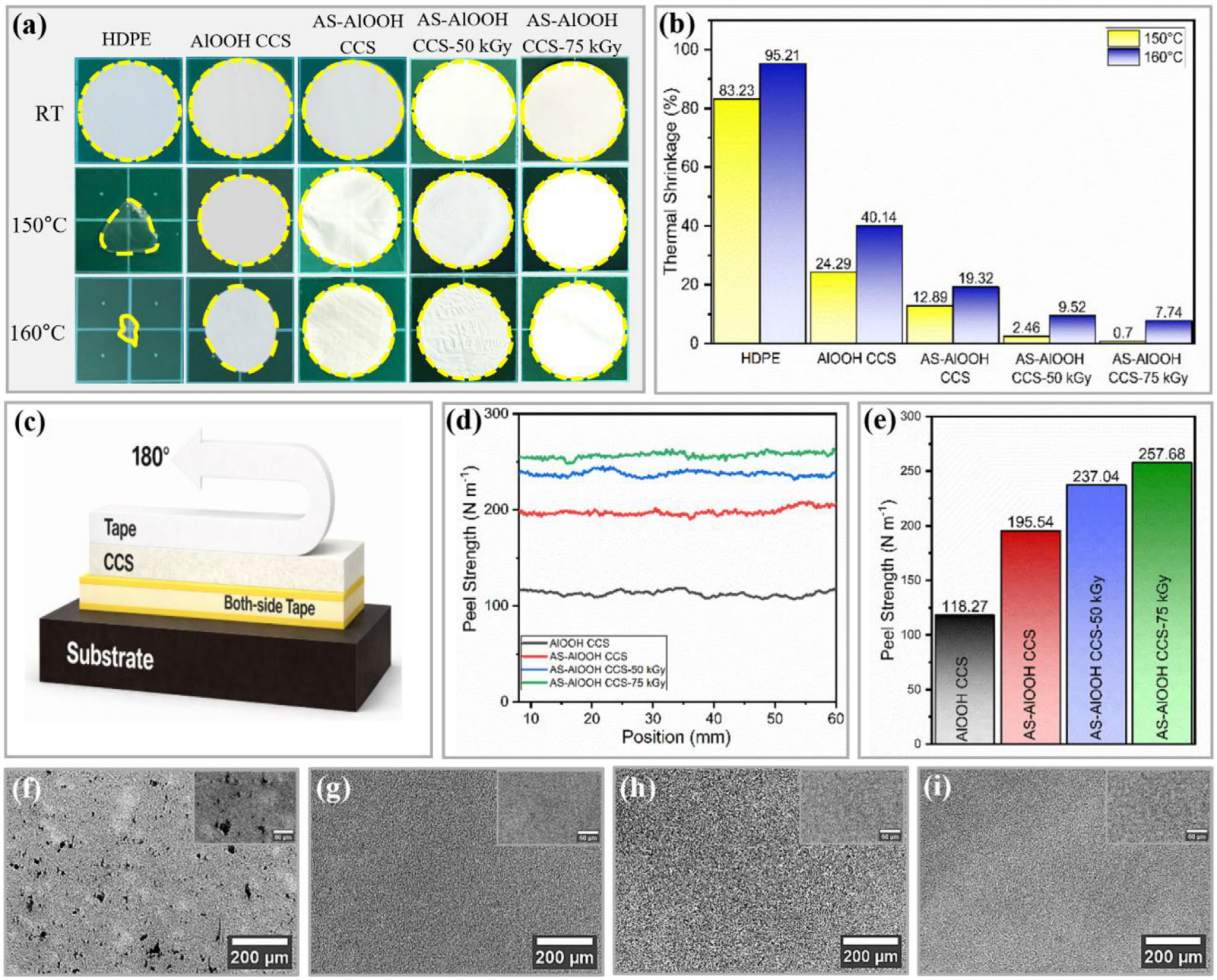
(a) Digital photographs of HDPE, AlOOH CCS, AS‐AlOOH CCS, AS‐AlOOH CCS‐50 kGy, and AS‐AlOOH CCS‐75 kGy separators at room temperature (RT) and after thermal treatment at 150°C and 160°C for 30 min. (b) Corresponding thermal shrinkage at 150°C and 160°C. (c) Schematic illustration of the 180° peel test configuration for evaluating adhesion between the ceramic coating and separator substrate. (d) Peel strength profiles as a function of position for AlOOH CCS, AS‐AlOOH CCS, AS‐AlOOH CCS‐50 kGy, and AS‐AlOOH CCS‐75 kGy. (e) Average peel strength values. (f–i) FESEM images of AlOOH CCS, AS‐AlOOH CCS, AS‐AlOOH CCS‐50 kGy, and AS‐AlOOH CCS‐75 kGy separator surfaces after peel testing.

The 180° peel strength measurement results (Figure 4c,d) further corroborate the interfacial strengthening behavior by directly probing the adhesion between the ceramic coating layer and the HDPE substrate. Figure 4e shows the peel strengths of the separators. The post‐peel test morphologies of the investigated separators are presented in Figure 4f–i. The AlOOH CCS separator exhibits a relatively low peel strength (118.27 N m^−1^), reflecting weak interfacial bonding primarily governed by physical contact and van der Waals interactions. The chemically inert HDPE surface lacks reactive functional groups for strong chemical coupling, leading to premature debonding and extensive coating removal after peeling, as observed in the post‐test morphology (Figure 4f). In contrast, the AS‐AlOOH CCS separator shows a significantly higher peel strength of 195.54 N m^−^^1^. This improvement arises from APTES‐mediated interfacial coupling, in which the protonated amine (–NH_3_^+^) groups on the AS‐AlOOH surface interact with the carboxyl (–COO^−^) groups of the PAA binder via ionic interactions, as supported by FTIR and XPS. These interactions strengthen cohesion within the ceramic–binder coating and, together with its improved uniformity and contact with the HDPE surface, increase the measured resistance to coating delamination. Similar improvements in coating adhesion have been reported for chemically crosslinked interfacial networks in ceramic‐coated separators, where enhanced chemical connectivity between inorganic particles and polymer binders significantly improves coating stability and mechanical robustness [28, 32, 53].

Electron beam (EB) irradiation further reinforces this interface by activating both the PAA binder and the HDPE substrate. High‐energy electrons generate radicals on the HDPE and PAA surfaces and induce crosslinking within the polymer matrix through radiation‐induced radical recombination reactions, similar to EB‐induced crosslinking of PAA binder reported in Li‐ion battery electrodes [51]. These processes may contribute to a chemically interconnected network across the ceramic–binder–HDPE junction, as illustrated in the interfacial mechanisms presented in Figure 2b. Such irradiation‐induced coupling increases the interfacial adhesion between the ceramic coating layer and the HDPE substrate. Consequently, the peel strength increases to 237.04 N m^−^^1^ for AS‐AlOOH CCS‐50 kGy. At 75 kGy (AS‐AlOOH CCS‐75 kGy), the peel strength further increases to 257.68 N m^−^^1^, consistent with the formation of a highly consolidated interface with almost no coating layer delamination (Figure 4i). Similar radiation‐induced crosslinking effects have been reported in EB‐irradiated polymer‐coated battery separators, where electron beam treatment generates crosslinked polymer networks that enhance coating adhesion and interfacial stability [50, 54]. This clear transition from weak physical adhesion (AlOOH CCS) to chemically bridged coupling (AS‐AlOOH CCS) and ultimately EB irradiation‐induced crosslinked anchoring shows that progressive interfacial connectivity contributes to coating durability in ceramic‐coated separators. Notably, the apparent divergence between tensile and peel responses at higher irradiation doses reflects a balance between interfacial reinforcement and bulk polymer extensibility. While peel resistance benefits from a dense crosslinked interphase that suppresses delamination of the coating layer, tensile performance mainly depends on the ability of the polymer matrix to accommodate large strains. Thus, moderate electron irradiation provides the most effective balance between interfacial adhesion and mechanical flexibility, whereas excessive irradiation preferentially enhances adhesion at the expense of bulk ductility. Together with the tensile and thermal shrinkage results, these findings demonstrate that APTES functionalization and controlled EB irradiation synergistically transform the ceramic‐coated separator into a mechanically integrated composite with strong interfacial adhesion and enhanced thermal stability. Such hierarchical reinforcement of the ceramic–polymer interface is expected to play a critical role in improving separator reliability and safety during Li‐ion battery operation.

The trends in Figure 5 indicate that separator surface chemistry plays a decisive role, alongside structural porosity, in governing electrolyte infiltration and ion transport. Although pristine HDPE has a high porosity (51.82%), its hydrophobic surface limits electrolyte spreading and penetration into the pore network, leading to incomplete wetting and increased ionic resistance. The pristine HDPE separator, consisting of nonpolar –CH_2_– chains, exhibits intrinsically low surface energy and limited affinity for polar carbonate‐based electrolytes. The inherent hydrophobic nature of polyolefin‐based separators, arising from the absence of polar functional groups along the backbone, is well recognized as a primary limitation that impedes uniform electrolyte distribution within the pore network [1, 2, 4, 55]. Consequently, the pristine separator exhibits a relatively high contact angle (∼36° at 1 s), and the digital photograph recorded after 60 s shows comparatively limited electrolyte spreading on its surface despite its high porosity. Accordingly, the electrolyte uptake remains relatively moderate at 92.3%. Representative electrochemical impedance spectra of the different separators are presented in Figure 5c, while the corresponding mean ionic conductivities, calculated from three independent measurements for each separator, are shown in Figure 5d. The individual bulk‐resistance values, calculated ionic conductivities, and associated statistical parameters are summarized in Table S3. The absence of polar interaction sites leads to incomplete electrolyte wetting within the porous network and increased ionic transport resistance, resulting in the highest bulk resistance of 3.59 ± 0.12 Ω, the lowest ionic conductivity of 0.166 ± 0.005 mS cm^−1^, and a relatively low Li^+^ transference number ($t_{Li^{+}}$≈ 0.43; Figure S6a). This behavior is consistent with previous reports showing that hydrophobic polyolefin separators exhibit limited electrolyte affinity and inferior ionic transport due to their nonpolar surfaces [4, 55, 56]. Introducing an AlOOH ceramic coating substantially improves electrolyte wettability. The AlOOH CCS separator exhibits a markedly reduced contact angle (26.11° at 1 s), and the corresponding photograph shows substantially greater electrolyte spreading across the coated surface after 60 s. Although the overall porosity decreases to 43.96% because of partial pore coverage by the ceramic layer, the electrolyte uptake increases to 116.84%. This enhancement arises from the abundant hydroxyl (–OH) groups on the AlOOH surface, which increase the surface free energy and promote capillary‐driven infiltration of the carbonate electrolyte through favorable polar and hydrogen‐bonding interactions. The increased electrolyte uptake therefore reflects improved electrolyte wettability, as the polar Al–OH and Al–O surface sites reduce the liquid‐solid contact angle and facilitate electrolyte penetration into the porous structure. As a result, the bulk resistance decreases to 2.97 ± 0.05 Ω, while the ionic conductivity increases to 0.261 ± 0.005 mS cm^−1^. In addition, interactions between the polar Al–OH/Al–O surface sites and electrolyte species may modify the local ionic environment and contribute to the modest increase in Li^+^ selectivity ($t_{Li^{+}}$≈ 0.54; Figure S6b), in agreement with previous reports on hydroxyl‐containing ceramic‐coated separators [29, 30]. Further enhancement is observed for the AS‐AlOOH CCS separator, in which APTES introduces surface –NH_2_ /–NH_3_^+^ groups that interact with carboxyl/carboxylate groups of the PAA binder through hydrogen bonding and acid‐base ionic interactions. The resulting polar AS‐AlOOH–polymer interphase promotes rapid electrolyte spreading and greater electrolyte uptake. Functional amine (–NH_2_/–NH_3_^+^) groups are known to enhance electrolyte infiltration and ion transport by increasing surface polarity and strengthening electrolyte‐separator interactions [57, 58, 59, 60]. Consequently, the electrolyte uptake increases to 143.82%, the bulk resistance decreases to 2.30 ± 0.06 Ω, and the ionic conductivity increases to 0.338 ± 0.009 mS cm^−1^. Electrostatic interactions between protonated amine (–NH_3_^+^) groups and PF_6_^−^ anions may restrict anion mobility, thereby contributing to the increase in $t_{Li^{+}}$ to ∼0.59 (Figure S6c). This proposed mechanism is consistent with recently reported anion‐regulating strategies involving functional separator coatings, in which interactions between anions and surface‐tethered functional groups increase the relative contribution of Li^+^ to the total ionic current [57, 58, 61, 62, 63]. Electron‐beam irradiation further modifies the polarity and chemical environment of the AS‐AlOOH CCS separators without substantially compromising their porous architecture. For the AS‐AlOOH CCS‐50 kGy separator, rapid and extensive electrolyte spreading is observed, and the electrolyte uptake increases to 147.62%. Consequently, the bulk resistance decreases further to 1.22 ± 0.06 Ω, the ionic conductivity increases to 0.635 ± 0.030 mS cm^−1^, while $t_{Li^{+}}$ rises to 0.66 (Figure S6d). EB irradiation modifies the interfacial chemical environment, as reflected by changes in the relative contributions of oxygen‐containing functionalities, including carbonyl and carboxyl groups, in the XPS spectra. These polar functionalities increase the surface energy and electrolyte affinity of the ceramic–polymer interphase through favorable hydrogen bonding and dipolar interactions with carbonate solvents. This behavior is consistent with previous reports on EB‐irradiated HDPE‐based separators [39, 42] and crosslinked polymer‐electrolyte systems, in which irradiation‐induced interfacial modification and network formation have been associated with improved ionic conductivity and Li^+^ selectivity [64].

**FIGURE 5:**
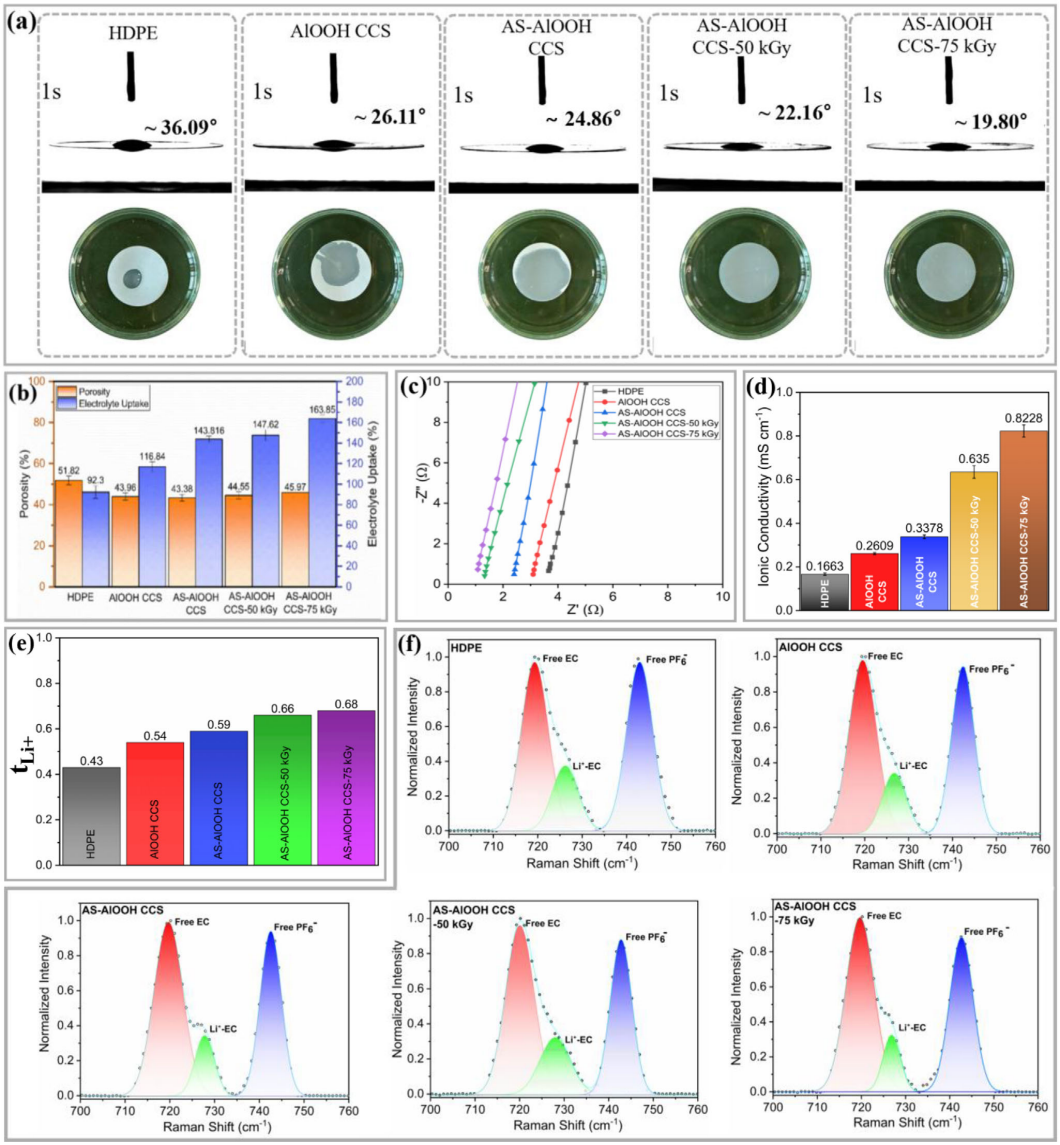
(a) Electrolyte contact‐angle images recorded at 1 s and digital photographs showing electrolyte spreading after 60 s on the HDPE, AlOOH CCS, AS‐AlOOH CCS, AS‐AlOOH CCS‐50 kGy, and AS‐AlOOH CCS‐75 kGy separators. (b) Porosity and electrolyte uptake of the separators. (c) Representative electrochemical impedance spectra of SS | electrolyte‐soaked separator | SS cells assembled with the separators. (d) Mean ionic conductivity and (e) Li^+^ transference number of the separators. (f) Deconvoluted Raman spectra of the electrolyte‐soaked separators in the 700–760 cm^−1^ region, showing the contributions of free EC, Li^+^‐coordinated EC, and free PF_6_^−^.

At 75 kGy, electrolyte uptake and ionic transport are enhanced further. The AS‐AlOOH CCS‐75 kGy separator exhibits the highest electrolyte uptake (163.85%), the lowest bulk resistance (0.943 ± 0.032 Ω), and the highest ionic conductivity of 0.823 ± 0.028 mS cm^−1^. Its Li^+^ transference number also increases to 0.68 (Figure S6e), establishing a monotonic rise in $t_{Li^{+}}$ across the separator series. The simultaneous increases in ionic conductivity and $t_{Li^{+}}$ are consistent with improved electrolyte infiltration and a more polar interfacial environment, together with the possible restriction of anion mobility, which increases the relative Li^+^ contribution to the total ionic current. The high electrolyte uptake maintains continuous electrolyte‐filled ion‐transport pathways, while interfacial chemical modifications resulting from EB‐initiated radical reactions enhance electrolyte affinity and contribute to reduced ionic‐transport resistance [10, 65].

To obtain complementary information on the local ionic environment, Raman spectra of the electrolyte‐soaked separators were deconvoluted over the 700–760 cm^−1^ region (Figure 5f). The bands at approximately 719, 728, and 742 cm^−1^ were assigned to free EC, Li^+^‐coordinated EC, and free PF_6_^−^, respectively [66]. The normalized Raman intensities and calculated intensity ratios are summarized in Table S4. The free PF_6_^−^/free EC intensity ratio (I_742_/I_719_) progressively decreases from 0.9911 for HDPE to 0.9635, 0.9473, 0.9167, and 0.8850 for AlOOH CCS, AS‐AlOOH CCS, AS‐AlOOH CCS‐50 kGy, and AS‐AlOOH CCS‐75 kGy, respectively. This trend indicates a progressively lower relative spectral contribution from free PF_6_^−^ and is consistent with modification of the local anionic environment through possible interactions with the Al–OH/Al–O sites of AlOOH, protonated –NH_3_^+^ groups introduced by APTES functionalization, and irradiation‐induced polar functionalities within the ceramic–polymer interphase. Meanwhile, the free EC/Li^+^‐coordinated EC intensity ratio (I_719_/I_728_) progressively increases from 2.6208 for HDPE to 2.8645, 2.8798, 2.9826, and 3.0788 for AlOOH CCS, AS‐AlOOH CCS, AS‐AlOOH CCS‐50 kGy, and AS‐AlOOH CCS‐75 kGy, respectively. This increase indicates a progressively lower relative spectral contribution from Li^+^‐coordinated EC and suggests modification of the local Li^+^ solvation environment toward a lower relative degree of EC coordination. This trend is consistent with modification of the local Li^+^‐EC coordination environment and may favor interfacial Li^+^ transport. Together with the possible restriction of PF_6_^−^ through interactions with polar surface functionalities, the altered solvation environment may increase the relative contribution of Li^+^ to the total ionic current. These systematic Raman changes are therefore consistent with the measured increase in $t_{Li^{+}}$ from 0.43 for HDPE to 0.54, 0.59, 0.66 and 0.68 for AlOOH CCS, AS‐AlOOH CCS, AS‐AlOOH CCS‐50 kGy, and AS‐AlOOH CCS‐75 kGy, respectively.

Overall, the progressive introduction of hydroxyl, amine, and irradiation‐induced oxygen‐containing functionalities transforms the separator surface from hydrophobic to highly electrolyte‐wettable, thereby improving electrolyte infiltration and ionic conductivity. The accompanying changes in the local PF_6_^−^ and Li^+^ solvation environments are consistent with the monotonic increase in $t_{Li^{+}}$ across the separator series. Among the investigated separators, AS‐AlOOH CCS‐75 kGy exhibits the most favorable overall ion‐transport properties, combining the highest electrolyte uptake, ionic conductivity, and Li^+^ transference number. This improved electrolyte affinity and Li^+^ selectivity is expected to alleviate concentration polarization and contribute to enhanced rate capability and cycling stability.

### 2.3 Electrochemical Performance

The practical electrochemical consequences of the progressively improved ion transport properties of the separators were subsequently evaluated in Li | LCO half‐cells assembled with HDPE, AlOOH CCS, AS‐AlOOH CCS, AS‐AlOOH CCS‐50 kGy, and AS‐AlOOH CCS‐75 kGy separators. Rate capability was assessed over C‐rates ranging from 0.5 to 20 C for cells assembled with the respective separators. The results are presented in Figure 6a, and the corresponding discharge capacities at 0.5 and 20 C are summarized in Table S5. At 0.5 C, the cells delivered discharge capacities of 123.92, 127.28, 130.80, 131.02, and 133.60 mAh g^−1^ for HDPE, AlOOH CCS, AS‐AlOOH CCS, AS‐AlOOH CCS‐50 kGy, and AS‐AlOOH CCS‐75 kGy, respectively. The comparable initial capacities across the separator series indicate that the surface modifications do not adversely affect the accessible capacity of the LCO cathode at low current density. The critical differentiation emerged under high‐rate operation. When the rate was increased to 20 C, the capacities decreased to 38.67, 56.11, 66.80, 99.90, and 102.63 mAh g^−1^, respectively. The cell assembled with pristine HDPE exhibited the lowest discharge capacity across all C‐rates, consistent with its limited electrolyte wettability, lower ionic conductivity, and lower Li^+^ transference number (Figure 5 and Figure S6). Its capacity retention of approximately 31% at 20 C relative to 0.5 C is consistent with greater concentration polarization, because limited electrolyte wetting and a lower Li^+^ transference number ($t_{Li^{+}}$) can produce a less uniform ion‐concentration profile across the separator under high Li^+^ flux [62]. The cell assembled with the AlOOH CCS separator showed improved rate capability, delivering 127.28 and 56.11 mAh g^−1^ at 0.5 C and 20 C, respectively, consistent with its enhanced electrolyte affinity and ionic transport [29, 30]. A further improvement was observed for AS‐AlOOH CCS, consistent with the increased electrolyte uptake, ionic conductivity, and Li^+^ transference number achieved through APTES functionalization. The improved high‐rate response is also consistent with electrostatic interactions between PF_6_^−^ and protonated amine (‐NH_3_^+^) groups, which may partially restrict anion mobility relative to Li^+^ [18, 21, 62], as discussed in the preceding section. The EB‐irradiated separators exhibited the best rate performance. In particular, AS‐AlOOH CCS‐50 kGy and AS‐AlOOH CCS‐75 kGy maintained high capacities of 99.90 and 102.63 mAh g^−1^ at 20 C, respectively, in line with their higher electrolyte uptake, lower bulk resistance, higher ionic conductivity, and higher Li^+^ transference numbers (Figure 5 and Figure S6). Notably, the cell assembled with AS‐AlOOH CCS‐75 kGy separator retained approximately 77% of its 0.5 C capacity at 20 C, whereas the cell assembled with HDPE retained approximately 31%, representing a 2.5‐fold improvement. This improvement is consistent with reduced concentration polarization arising from the combined increases in electrolyte affinity, ionic conductivity, and Li^+^ transport, which mitigate ion‐concentration gradients across the separator during high‐rate operation. These improvements collectively demonstrate the electrochemical benefits of the hierarchically engineered ceramic‐polymer interface. When the rate was returned to 0.5 C, the capacity nearly recovered its initial value. This near‐complete recovery indicates that the high‐rate capacity attenuation was governed predominantly by reversible kinetic polarization and demonstrates the ability of the engineered separators to sustain high‐current operation without appreciable irreversible performance loss during the rate test [10, 67].

**FIGURE 6:**
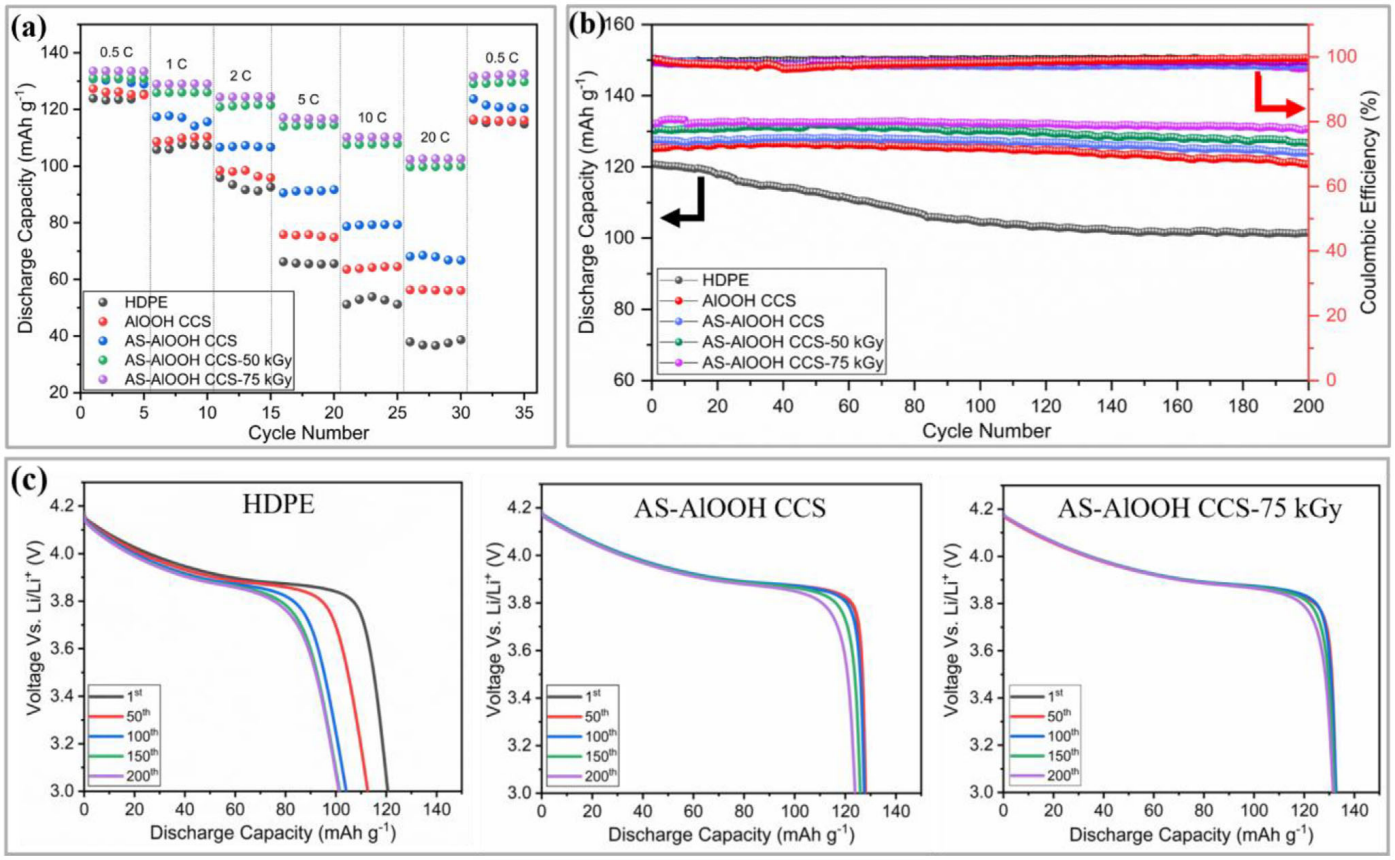
(a) Rate capability of cells at C‐rates ranging from 0.5 to 20 C. (b) Cycling performance and coulombic efficiency at 0.5 C for 200 cycles. (c) Discharge voltage profiles of cells assembled with HDPE, AS‐AlOOH CCS, and AS‐AlOOH CCS‐75 kGy at selected cycle numbers.

Cycling performance was evaluated at 0.5 C over 200 cycles to compare the long‐term capacity retention of cells assembled with the different separators, as presented in Figure 6b. The discharge capacities at the first and 200th cycles are summarized in Table S6. The cell assembled with pristine HDPE exhibited the lowest capacity retention, with its discharge capacity decreasing from 120.69 mAh g^−1^ at the first cycle to 101.51 mAh g^−1^ at the 200th cycle, corresponding to a retention of 84.11%. This capacity fading is consistent with the low electrolyte affinity and limited ion‐transport properties of the HDPE separator, as well as the pronounced increase in charge‐transfer resistance (R_ct_) observed after cycling (Figure 7b) [68]. Introducing the AlOOH ceramic coating (AlOOH CCS) improved the capacity retention to 94.96%, maintaining a discharge capacity of 118.90 mAh g^−^^1^ at the 200th cycle. This improvement is consistent with the enhanced electrolyte affinity and ionic transport enabled by the polar ceramic surface. The AS‐AlOOH CCS‐based cell exhibited a further improvement, maintaining 123.88 mAh g^−1^ at 200th cycle with a capacity retention of 97.12%. This behavior is consistent with the enhanced electrolyte affinity, ionic conductivity, and Li^+^ transference number achieved through APTES functionalization. Protonated amine (–NH_3_^+^) groups may interact electrostatically with PF_6_^−^ anions and modify their local coordination environment, thereby reducing their effective mobility relative to Li^+^ and alleviating concentration polarization. Similar effects have been reported for amine‐enriched separators [62]. In the present system, the decreasing free PF_6_^−^/free EC Raman intensity ratio supports a change in the local PF_6_^−^ environment. Together with the increased Li^+^ transference number, this result is consistent with stronger separator–anion interactions and a greater relative contribution of Li^+^ to ionic conduction, promoting more uniform Li^+^ flux during prolonged cycling. The EB‐irradiated separators exhibited the highest capacity retention. AS‐AlOOH CCS‐50 kGy maintained 126.63 mAh g^−^^1^ at 200th cycle with 97.20% retention, whereas AS‐AlOOH CCS‐75 kGy maintained 130.78 mAh g^−^^1^ with 98.97% retention. This improved durability is attributed to the combined effects of enhanced coating integrity and favorable ion transport. EB‐induced interfacial reorganization can strengthen particle–binder cohesion and help preserve coating adhesion during prolonged electrolyte exposure and cycling. The resulting stable, electrolyte‐affinitive coating helps maintain continuous ion‐transport pathways and homogeneous Li^+^ flux, thereby mitigating local current‐density variations, concentration polarization, and the development of resistive electrode–electrolyte interfaces [35, 62, 64, 67, 69]. Accordingly, the HDPE‐based cell exhibits progressively increased polarization and capacity loss (Figure 6c), whereas the AS‐AlOOH CCS‐75 kGy‐based cell shows closely overlapping discharge profiles with minimal polarization shift over 200 cycles (Figure 6c), indicating better preservation of Li^+^ transport and overall cell kinetics.

**FIGURE 7:**
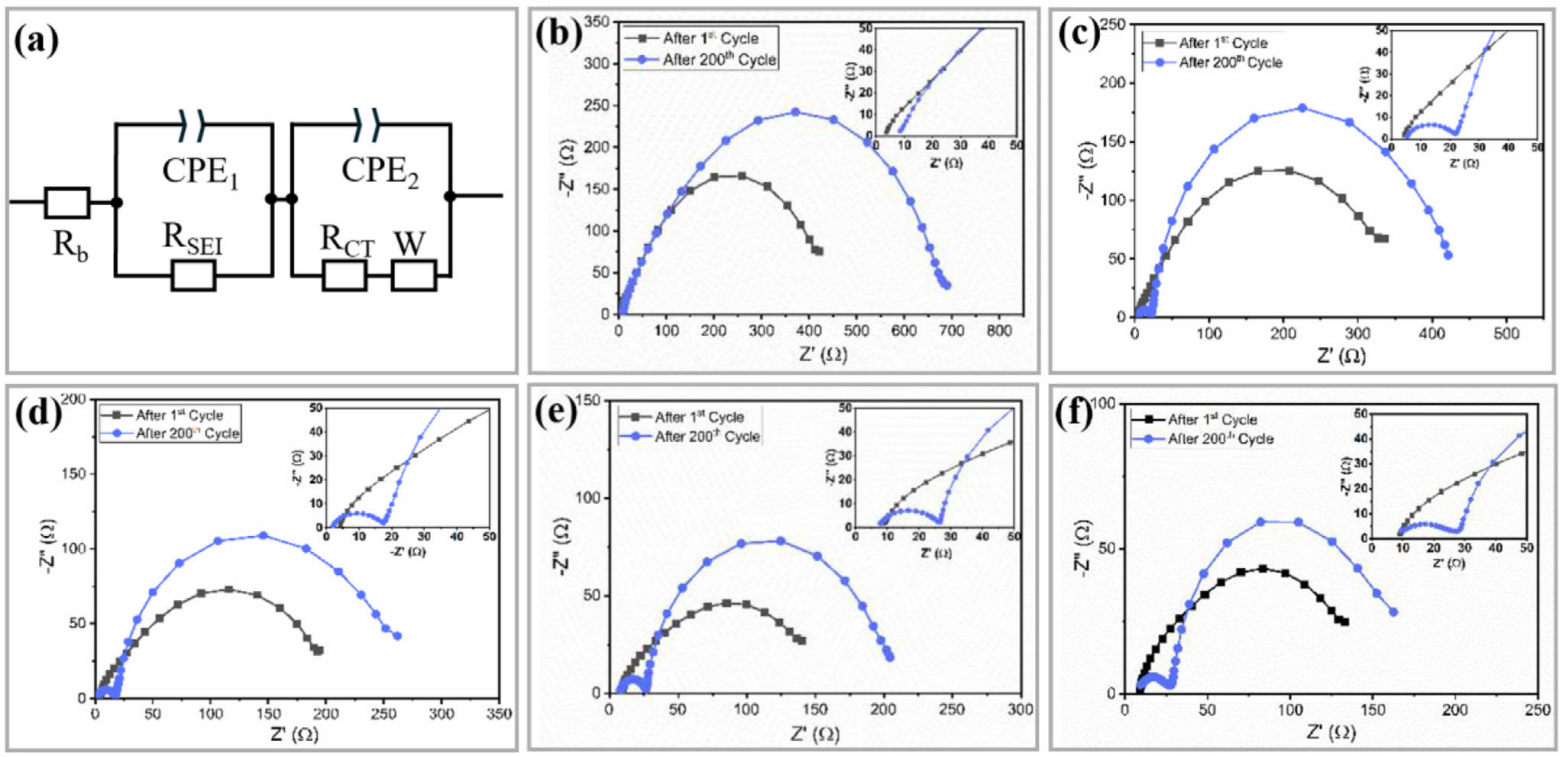
(a) Equivalent circuit model used for fitting electrochemical impedance spectra. Nyquist plots of Li | separator + electrolyte | LCO cells with different separators measured after the first and 200th cycles: (b) HDPE, (c) AlOOH CCS, (d) AS‐AlOOH CCS, (e) AS‐AlOOH CCS‐50 kGy, and (f) AS‐AlOOH CCS‐75 kGy. Insets show enlarged high‐frequency regions.

To further elucidate the interfacial charge‐transfer characteristics governing the electrochemical performance, EIS measurements were conducted after the first and 200th cycle. The corresponding Nyquist plots and equivalent circuit model are presented in Figure 7a–f, and the fitted impedance parameters are summarized in Table S7. The cell with HDPE separator exhibited the highest charge‐transfer resistance (R_ct_), increasing markedly from 436 Ω after the first cycle to 691 Ω after the 200th cycle (Figure 7b). This pronounced increase indicates progressively hindered charge‐transfer kinetics and is consistent with the poor electrolyte wettability and limited ion‐transport properties of the hydrophobic HDPE separator. Introducing the AlOOH ceramic coating lowers​ R_ct_ to 360 Ω after the first cycle and 415 Ω after 200th cycle (Figure 7c), consistent with improved electrolyte infiltration and more homogeneous Li^+^ transport enabled by the polar ceramic surface.

For AS‐AlOOH CCS, R_ct_ increases from 192 to 251 Ω (Figure 7d), reflecting the improved electrolyte affinity, ionic conductivity, and Li^+^ transference number achieved through APTES functionalization. The amine‐containing surface may regulate the local anion environment and promote more homogeneous Li^+^ flux, thereby mitigating concentration gradients and limiting the development of a heterogeneous resistive interface [62]. The EB‐irradiated separators exhibit the lowest R_ct_ values and the smallest increases during cycling: from 145 to 183 Ω for AS‐AlOOH CCS‐50 kGy and from 127 to 142 Ω for AS‐AlOOH CCS‐75 kGy (Figure 7e,f). These results indicate that EB irradiation helps preserve coating integrity, electrolyte distribution, and ion‐transport pathways during prolonged cycling. Overall, the reduced R_ct_ and suppressed impedance growth demonstrate improved charge‐transfer kinetics and long‐term interfacial stability, consistent with the enhanced rate capability and cycling durability observed in Figure 6.

A radar plot qualitatively summarizing the key physicochemical and electrochemical properties of the separators under study is presented in Figure 8a, comparing tensile strength, dimensional stability (150°C, 30 min), peel strength, electrolyte uptake, ionic conductivity, and capacity retention at the 200th cycle. The pristine HDPE separator exhibits the smallest radar area, reflecting its limited electrolyte affinity and lower electrochemical performance across all selected metrics.

**FIGURE 8:**
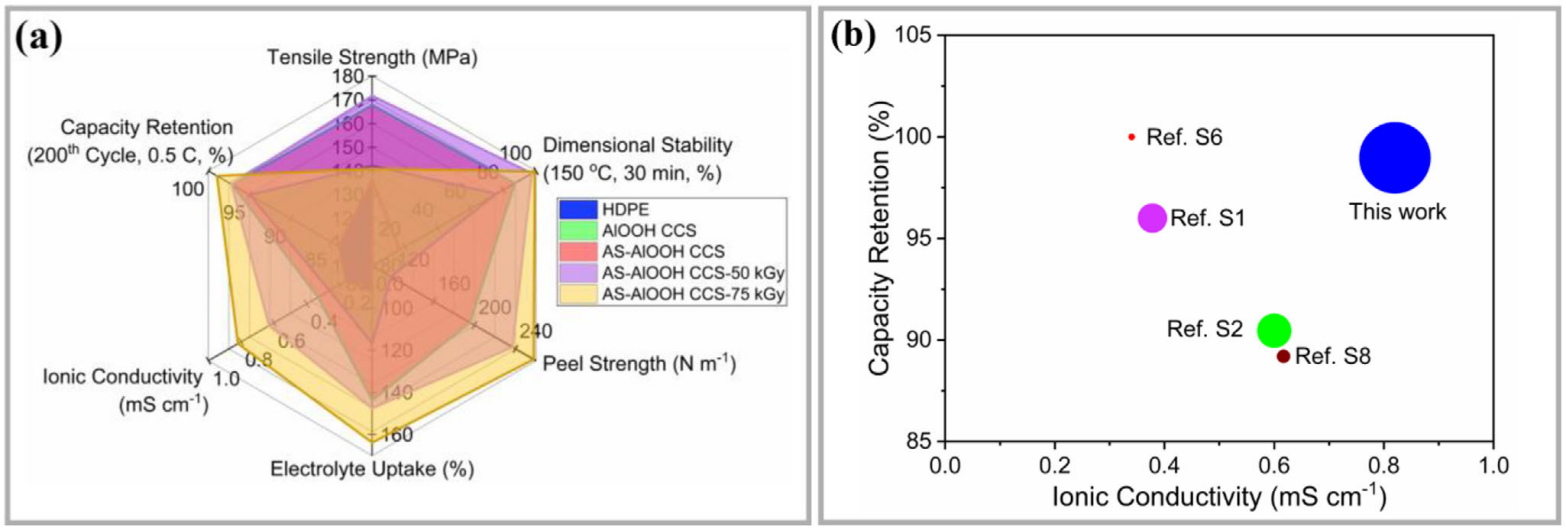
(a) Radar comparison of mechanical, thermal, and electrochemical properties of HDPE, AlOOH CCS, AS‐AlOOH CCS, and EB‐treated separators, highlighting the synergistic performance enhancement through APTES functionalization and EB irradiation. (b) Comparison of ionic conductivity, capacity retention, and adhesion strength (proportional to circle area) with previously reported PAA‐based ceramic‐coated separators.

Each successive level of interfacial engineering–AlOOH coating, APTES functionalized AlOOH coating, and subsequent EB irradiation–systematically expands the radar profile, with AS‐AlOOH CCS‐75 kGy displaying the largest and most balanced area. Consistent with these findings, Figure 8b benchmarks ionic conductivity, capacity retention, and adhesion strength against previously reported PAA‐based ceramic‐coated separators, demonstrating favorable combined performance relative to the selected examples summarized in Table S8. The simultaneous improvement in mechanical robustness, interfacial adhesion, thermal stability, and electrochemical performance demonstrates the benefit of the hierarchical interfacial engineering strategy, because these properties can be difficult to optimize concurrently [1, 2, 3, 9, 10, 11, 33, 55, 56, 69]. Collectively, these results establish that integrating ceramic coating, silane‐mediated interfacial coupling, and EB irradiation‐induced interfacial modification and potential radical‐mediated coupling produces a separator architecture with a favorable balance of mechanical, thermal, and electrochemical properties, providing a promising approach for the design of advanced lithium‐ion batteries.

## 3 Conclusion

In this work, a hierarchical interfacial engineering strategy integrating APTES‐functionalized AlOOH nanoparticles with controlled electron‐beam irradiation is developed to construct mechanically robust and thermally stable ceramic‐coated HDPE separators. APTES functionalization enhanced ceramic–binder coupling through Al─O─Si bonds and amine–carboxylate interactions, leading to uniform coating and improved interfacial adhesion. Subsequent electron‐beam irradiation produced chemical changes and network formation, consistent with radical‐mediated reactions, within the ceramic–polymer framework, significantly enhancing mechanical strength, coating integrity, and thermal stability. The optimized separator exhibited excellent electrolyte wettability, high ionic conductivity (∼0.82 mS cm^−1^), and an increased Li^+^ transference number (∼0.68). Consequently, Li ∣ LCO cells assembled with the engineered separators demonstrated outstanding rate capability and nearly 99% capacity retention after 200 cycles. These findings highlight hierarchical interface engineering as an effective strategy for developing thermally stable and high‐performance separators for advanced lithium‐ion batteries.

## 4 Experimental Section

### 4.1 Materials

High‐density polyethylene (HDPE) separators and boehmite (AlOOH, ∼500 nm) nanoparticles were supplied by LG Energy Solutions, Daejeon, South Korea. (3‐aminopropyl)triethoxysilane (APTES), poly(acrylic acid) (PAA, M_w_ = 500,000), and 2,4,7,9‐Tetramethyl‐5‐decyne‐4,7‐diol (TMDD) ethoxylate were purchased from Sigma‐Aldrich (South Korea). The liquid electrolyte, 1 m LiPF_6_ in ethylene carbonate/diethyl carbonate/ethyl methyl carbonate (1:1:1, v/v/v) containing 3 wt.% fluoroethylene carbonate, was supplied by Donghwa Electrolyte Co. Super P (Imerys Co.), N‐methyl‐2‐pyrrolidone (NMP; Jaewon Industrial Co.), lithium cobalt oxide (Cosmo AM&T Co.), Al foil (Dongil Aluminum Co.), and Li metal (Honjo Metal Co.) were used as received.

### 4.2 APTES Functionalization of Boehmite (AlOOH) Nanoparticles

APTES functionalization of AlOOH nanoparticles was carried out via a hydrolysis–condensation route. Briefly, 3 g of pre‐dried AlOOH nanoparticles were dispersed in 100 mL of ethanol under mechanical stirring, followed by ultrasonication to ensure uniform dispersion. APTES hydrolysis was performed by adding 4 mL of APTES to 100 mL of an EtOH–water solution (98:2, v/v), adjusting the pH to ∼6 with 1 m HCl, and stirring for 30 min. The hydrolyzed APTES solution was then slowly added to the AlOOH dispersion, and the mixture was stirred continuously at 110°C for 12 h to promote condensation of silanol groups with surface hydroxyl groups. The resulting APTES‐functionalized AlOOH nanoparticles were collected by centrifugation (10 000 rpm, 10 min), washed three times with ethanol to remove residual silane, and dried at 100°C. The APTES‐grafted AlOOH nanoparticles were hereafter referred to as AS‐AlOOH.

### 4.3 Preparation of Coating Slurry and Ceramic Coated Separators (CCS)

The coating slurry was prepared by dispersing AlOOH or APTES‐functionalized AlOOH (AS‐AlOOH) nanoparticles in deionized water, followed by the addition of a wetting agent (TMDD). The suspension was stirred (6 h) and ultrasonicated (20 min) to ensure uniform dispersion. Subsequently, a 10 wt% PAA solution was introduced, maintaining an AlOOH: PAA ratio of 90:10 (wt%). The mixture was further homogenized by sequential stirring (6 h), ultrasonication (20 min), and an additional 6 h of stirring to obtain a stable slurry. The resulting slurry was coated on both sides of an HDPE separator using an automatic film applicator and dried at 80°C for 12 h to ensure complete solvent removal and binder consolidation. The final ceramic‐coated separators had a total thickness of ∼13 µm, with a uniform ceramic coating of ∼1.5 µm on each side. Electron‐beam (EB) irradiation was subsequently applied at doses of 25, 50, 75, and 100 kGy using an electron accelerator (EB Tech Co., Daejeon, Republic of Korea) to investigate dose‐dependent interfacial and structural evolution in AS‐AlOOH CCS. The samples are denoted as HDPE, AlOOH CCS, AS‐AlOOH CCS, and AS‐AlOOH CCS‐X, where X represents the EB dose.

### 4.4 Characterization

Fourier transform infrared spectroscopy (FTIR, Nicolet iS50, Thermo Fisher Scientific), transmission electron microscopy (TEM) with energy‐dispersive X‐ray spectroscopy (EDS) mapping (Talos F200X G2, Thermo Fisher), and thermogravimetric analysis (TGA, STA 449 F3 Jupiter, NETZSCH) were used to confirm APTES functionalization of AlOOH nanoparticles. The surface and cross‐sectional morphology of the separators were examined using field‐emission scanning electron microscopy (FESEM, SU5000, Hitachi) equipped with an EDS detector. Cross‐sections of the separators were prepared via cryo‐ion milling (ArBlade5000, Hitachi). FTIR analysis was also conducted to identify functional groups and interactions among the ceramic nanoparticles, PAA binder, and HDPE substrate. X‐ray photoelectron spectroscopy (XPS) measurements were performed using a Nexsa G2 XPS system (Thermo Scientific, USA) to investigate the surface elemental composition and chemical states of the separators. Raman spectroscopy was performed using an inVia Qontor Raman spectrometer (Renishaw, UK). The electrolyte‐soaked separators were analyzed, and the spectra were baseline‐corrected and normalized. The mechanical properties were measured using a universal testing machine (Instron 5848, Instron, USA) with a sample width of 2 cm, gauge length of 5 cm, and crosshead speed of 50 mm min^−1^. The adhesion strength of the coating layer was measured using 180° peel test in accordance with ASTM D3330. Thermal shrinkage was measured by heating the separators at selected temperatures for 30 min and recording the area before and after thermal exposure. The thermal shrinkage was determined by heating separators at selected temperatures for 30 min, and applying Equation (1).

(1) $$Thermal\;Shrinkage\left(\%\right)=\frac{A_{0}-A}{A_{0}}\times 100$$

where *A_0_* and *A* represent the separator area before and after heat exposure, respectively.

Porosity of the separators was measured using the hexadecane immersion method. The dry weight (*W_dry_*) was recorded, followed by immersion in hexadecane for 2 h. After wiping the excess liquid, the wet weight (*W_wet_*) was measured, and the porosity was calculated using Equation (2)

(2) $$Porosity\left(\%\right)=\frac{W_{wet}-W_{dry}}{\rho_{hex}\times V_{sep}}\times 100$$

where ρ_*hex*_ is the density of hexadecane and *V_sep_* is the separator volume.

Electrolyte uptake was determined from the mass change before and after electrolyte absorption. The initial dry weight (*W_i_*) was recorded, followed by immersion in liquid electrolyte for 2 h to ensure full saturation. After removing excess surface electrolyte, the final weight (*W_f_*) was measured. Electrolyte uptake was calculated using Equation (3)

(3) $$Electrolyte\;Uptake\left(\%\right)=\frac{W_{f}-W_{i}}{W_{i}}\times 100$$

Electrolyte wettability was evaluated using a contact angle goniometer. The apparent contact angle was recorded 1 s after droplet electrolyte deposition, and electrolyte spreading was photographed 60 s after dropping 10 µL of electrolyte.

Ionic conductivity was measured using AC impedance spectroscopy (SP‐200, BioLogic, France) over 100 kHz to 10 Hz with a 10 mV amplitude. Electrolyte‐soaked separators were sandwiched between two stainless steel electrodes to assemble the test cells. Three independently assembled cells were measured for each separator, and results are reported as the mean ± standard deviation. The ionic conductivity (σ) was calculated using Equation (4).

(4) $$\sigma =\frac{d}{R_{b}\times S}\;$$

where *d* is the separator thickness, *R_b_* is the bulk resistance, and *S* is the effective electrode–separator contact area.

The Li^+^ transference number ($t_{Li^{+}}$) was determined using the Bruce‐Vincent‐Evans method in Li || electrolyte‐soaked separator || Li cell configuration. The Li^+^ transference number was calculated using Equation (5).

(5) $$t_{Li^{+}}=\frac{I_{s}\left(\Delta V-I_{0}R_{0}\right)}{I_{0}\left(\Delta V-I_{s}R_{s}\right)}$$

where *ΔV* is the applied voltage, *I_0_* and *I_s_* are the initial and steady state currents, and *R_0_* and *R_s_* are the interfacial resistances before and after polarization.

Electrochemical performance was evaluated using CR2032 coin cells with LCO cathodes and lithium‐metal anodes. Cathodes were prepared from LCO, Super P, and PVDF (8:1:1 by weight) and cast onto Al foil at a loading of ∼2 mg cm^−2^. Rate capability was tested from 0.5 to 20 C, and cycling stability was assessed at 0.5 C for 200 cycles using a battery tester (WBCS 3000L32, WonAtech, Korea). Cell resistances after the first and 200th cycles were analyzed by AC impedance spectroscopy (100 kHz–100 mHz, 10 mV amplitude).

## Conflicts of Interest

The authors declare no conflicts of interest.

## Supporting information




**Supporting File**: smll75250‐sup‐0001‐SuppMat.docx.

## Data Availability

The data that support the findings of this study are available from the corresponding author upon reasonable request.
