# Supplementary material for: Hierarchical Interfacial Engineering of Boehmite‐Coated Separators via Silane Functionalization and Electron‐Beam Irradiation
Source: Small. 2026 Aug 16;22(59):e75250. doi: 10.1002/smll.75250 (PMC13690611; doi:10.1002/smll.75250)
Supplement: Supplementary file 1 [file SMLL-22-e75250-s001.docx]

Supporting Information

**Hierarchical Interfacial Engineering of Boehmite-Coated Separators via Silane Functionalization and Electron-Beam Irradiation**

*Md Amir Sohel, Sungwoo Kim, Seunguk Cheon, Sung Oh Cho^*^*

Department of Nuclear and Quantum Engineering, Korea Advanced Institute of Science and Technology (KAIST), 291 Daehak-ro, Yuseong-gu, Daejeon 34141, Republic of Korea

E-mail: [socho@kaist.ac.kr](mailto:socho@kaist.ac.kr) (S. O. Cho)

1. **Supplementary Figures**

Figure S1. TGA results of AlOOH and AS-AlOOH

Figure S2. EDS mapping of C, O, Al and Si in AS-AlOOH CCS

Figure S3. (a) FTIR spectra of pristine and EB irradiated PAA film. (b) Gel fraction of PAA film as a function EB dose.

Figure S4. (a) Stress–strain curves of AS-AlOOH CCS separators with varying EB doses. (b) Tensile strength and elongation of HDPE, AlOOH CCS, AS-AlOOH CCS, and EB-treated samples. (c) Stress–strain curves of pristine HDPE under different EB doses. (d) Corresponding tensile strength and elongation of pristine and irradiated HDPE.

Figure S5. (a) Photographs of AS-AlOOH CCS separators with varying EB doses after thermal exposure at 150 and 160 °C for 30 min. (b) Corresponding thermal shrinkage values.

Figure S6. Li⁺ transference number $\left( t_{{Li}^{+}} \right)$ evaluation by the Bruce–Vincent method. Chronoamperometric responses under constant DC polarization for cells employing (a) HDPE, (b) AlOOH CCS, (c) AS-AlOOH CCS, (d) AS-AlOOH CCS-50 kGy, and AS-AlOOH CCS-75 kGy separators. Insets show the corresponding impedance spectra before and after polarization.

Figure S7. (a-e) Discharge voltage profiles of the cells with (a) HDPE, (b) AlOOH CCS, (c) AS-AlOOH CCS, (d) AS-AlOOH CCS-50 kGy and (e) AS-AlOOH CCS-75 kGy separators at different cycle numbers.

.

**2. Supplementary Tables**

Table S1. Discharge capacities of cells assembled with different separators at 0.5 and 20 C rates.

| Sample | Capacity (mAh g^-1^) | | Retention (%) |
| --- | --- | --- | --- |
|  | At 0.5 C | At 20 C |  |
| **HDPE** | 123.92 | 38.67 | 31.20 |
| **AlOOH CCS** | 127.28 | 56.11 | 44.08 |
| **AS-AlOOH CCS** | 130.80 | 66.80 | 51.07 |
| **AS-AlOOH CCS-50 kGy** | 131.02 | 99.90 | 76.21 |
| **AS-AlOOH CCS-75 kGy** | 133.60 | 102.62 | 76.81 |

Table S2. Discharge capacities of cells assembled with different separators at 1^st^ cycle and 200 cycle discharging at 0.5 C rate.

| Sample | Capacity (mAh g^-1^) | | Retention (%) |
| --- | --- | --- | --- |
|  | 1^st^ Cycle | 200^th^ Cycle |  |
| **HDPE** | 120.69 | 101.51 | 84.11 |
| **AlOOH CCS** | 125.21 | 118.90 | 94.96 |
| **AS-AlOOH CCS** | 127.55 | 123.88 | 97.12 |
| **AS-AlOOH CCS-50 kGy** | 130.27 | 126.63 | 97.20 |
| **AS-AlOOH CCS-75 kGy** | 132.14 | 130.78 | 98.97 |

Table S3. Electrochemical impedance values extracted from Nyquist plots of cells assembled with different separators measured after the 1^st^ and 200^th^ charge–discharge cycles.

| Sample | | R_b_ (Ω) | R_SEI_(Ω) | R_ct_ (Ω) | R_total_(Ω) |
| --- | --- | --- | --- | --- | --- |
| **HDPE** | 1^st^ Cycle | 3.1 | 15 | 436 | 454.1 |
|  | 200^th^ Cycle | 6.2 | 20 | 691 | 717.2 |
| **AlOOH CCS** | 1^st^ Cycle | 3.4 | 6 | 360 | 369.4 |
|  | 200^th^ Cycle | 3.6 | 16 | 415 | 434.6 |
| **AS-AlOOH CCS** | 1^st^ Cycle | 3.3 | 14 | 192 | 209.3 |
|  | 200^th^ Cycle | 2.1 | 16 | 251 | 269.1 |
| **AS-AlOOH CCS-50-kGy** | 1^st^ Cycle | 6.9 | 11 | 145 | 162.9 |
|  | 200^th^ Cycle | 6.3 | 19 | 183 | 209.3 |
| **AS-AlOOH CCS-75 kGy** | 1^st^ Cycle | 6.5 | 20 | 127 | 153.5 |
|  | 200^th^ Cycle | 5.3 | 24 | 142 | 171.3 |

Table S4. Comparison of key performance parameters achieved in this work with literature reports on PAA-based binders for ceramic-coated separators (CCSs).

| Substrate Separator (thickness in µm) | Binder | Ceramic | Thickness (µm) | Adhesion (N m^-1^) | Thermal shrinkage | Ionic conductivity (mS cm^-1^) | Cycling stability (retention, cycle number) | Refs. |
| --- | --- | --- | --- | --- | --- | --- | --- | --- |
| PE (not reported) | Pn-PPA | Al_2_O_3_ | 2 | 105.9 | 0% at 130°C | 0.378 | 96%, after 200 cycle (LFP\|\|Li) | [1] |
| PE (20) | APEG grafted PAA (PAA-APEG) | Al_2_O_3_ | Not reported | 124.3 | Not reported | 0.6 | 90.46% after 300 cycle (LFP\|\|Li) | [2] |
| PE (12) | PAA | Al_2_O_3_ | 4 | Not reported | 0 at 130°C | 0.4 | 92.3% after100 cycle (LCO\|\|Li) | [3] |
| PE (14) | PAA | ZrO_2_ | Not reported | Not reported | 78% at 140°C | 0.51 | 85.1% after 100 cycle | [4] |
| PE (16) | PAALi | Al_2_O_3_ | 3 | Not reported | 0% at 140°C | 0.34 | 90.2% after 180 cycle ((LCO\|\|Li) | [5] |
| PE (not reported) | P(AA-THFA)/GPTMS crosslinked | AlOOH | 2 | 24 | 0% at 150 °C | 0.34 | 100% after 450 cycle (LFP\|\|Li) | [6] |
| PE (16) | PAA | SiO_2_ | 4 | Not reported | Severe shrinkage at 110 °C | Not reported | 82.9 % after 600 cycle (NMC 811\|\|Li) | [7] |
| PE (9) | PAA | PDA treated  Mg (OH)_2_ | 6 | 49.10 | 0% at 160°C | 0.617 | 89.2% after 400 cycle (NMC811\|\|LI)/ | [8] |
| **PE (10)** | **PAA (EB crosslinked)** | **APTES treated AlOOH** | **1.5 um on each side** | **257.68** | **0.70% (at 150°C), 7.74% (at 160°C)** | **5.42** | **98.97% after 200 cycles (LCO\|\|Li)** | **This work** |

**References**

1.	A. Q. Thang, Thang, Y. Shen, Z. Shi, G. Yao, S.Y. Wong, Z. Liu, Q. Yan, Partially Neutralized Polyacrylic Acid as an Efficient Binder for Aqueous Ceramic-Coated Separators for Lithium-Ion Batteries, *Chem. Asian J.* 18 (2023) e202300538. https://doi.org/10.1002/asia.202300538.

2.	N. Dang, J. Mao, Y. Mao, W. Yi, D. Li, T. Cheng, L. He, J. Deng, Z. Zhao, T. Zhao, B. Chen, Functionalized Polyethylene Separators with Efficient Li-Ion Transport Rate for Fast-Charging Li-Ion Batteries, *ACS Appl. Mater. Interfaces.* 17 (2025) 2169–2179. <https://doi.org/10.1021/acsami.4c15898>.

3.	Q. Wang, J. Yang, Z. Wang, L. Shi, S. Yuan,Wang, Dual-Scale Al2O3 Particles Coating for High-Performance Separator and Lithium Metal Anode, *Energy Technol*. 8 (2020). 8(5) 1901429. https://doi.org/10.1002/ente.201901429.

4.	W. Xu, Z. Wang. L. Shi, Y. Ma, S. Yaun, L. Sun, Y. Zhao, M. Zhang, J. Zhu, Layer-by-Layer Deposition of Organic–Inorganic Hybrid Multilayer on Microporous Polyethylene Separator to Enhance the Electrochemical Performance of Lithium-Ion Battery, *ACS Appl. Mater. Interfaces*. 7 (2015) 20678–20686. <https://doi.org/10.1021/acsami.5b05457>.

5.	R. Xu, L. Sheng, H. Gong, Y. Kong, Y. Yang, M. Li, Y. Bai, S. Song, G. Liu, T. Wang, X. Huang, J. He, High-Performance Al2O3 /PAALi Composite Separator Prepared by Water-Based Slurry for High-Power Density Lithium-Based Battery, *Adv. Eng. Mate*r. 23 (2021) 2001009. https://doi.org/10.1002/adem.202001009.

6.	H. Zhu, B. Dong, X. Cai, L. Xi, P. Zhang, Y. Hao, S. Chen, R. Zeng, Zhu, H., In situ covalent crosslinking strategy to construct highly stable composite separators for lithium-ion batteries, *Chem. Eng. J*. 488 (2024) 151120. <https://doi.org/10.1016/j.cej.2024.151120>.

7.	L. Tam, Y. Sun, C. Wei, Y. Tao, Y. Tian, Y. An, Y. Zhang, S. Xiong, J. Feng, Design of Robust, Lithiophilic, and Flexible Inorganic-Polymer Protective Layer by Separator Engineering Enables Dendrite-Free Lithium Metal Batteries with LiNi0.8Mn0.1Co0.1O2 Cathode, *Small*. 17 (2021) 2007717. https://doi.org/10.1002/smll.202007717.

8.	Y. Roh, D. Jin, E. Kim, S. Byun, Y. -S. Lee, M. _H. Ryou, Y. M. Lee, Highly improved thermal stability of the ceramic coating layer on the polyethylene separator via chemical crosslinking between ceramic particles and polymeric binders, *Chem. Eng. J*. 433 (2022) 134501. <https://doi.org/10.1016/j.cej.2022.134501>.
